# Designer amphiphilic helical peptide-decorated nanomicelles enable simultaneous inflammation control and triple-destruction of bacteria for treating bacterial pneumonia and sepsis

**DOI:** 10.7150/thno.110538

**Published:** 2025-08-16

**Authors:** Sixia Liu, Rui Wang, Lian Li, Xiaohuan Wang, Jiameng Gong, Xingzu Liu, Zichen Song, Liya Sun, Xiali Liu, Wen Ning, Yuanlin Song, Shan-Yu Fung, Hong Yang

**Affiliations:** 1 Tianjin Key Laboratory of Inflammation Biology, The Province and Ministry Co-Sponsored Collaborative Innovation Center for Medical Epigenetics, Department of Pharmacology, School of Basic Medical Sciences, Intensive Care Unit of the Second Hospital, Tianjin Medical University, Tianjin 300070, China.; 2 State Key Laboratory of Medicinal Chemical Biology, College of Life Sciences, Tianjin Key Laboratory of Protein Sciences, Nankai University, Tianjin 300071, China.; 3 Department of Laboratory Medicine, Zhongshan Hospital, Fudan University, Shanghai 200032, China.; 4 Department of Anesthesia, Tianjin Institute of Anesthesiology, Tianjin Medical University General Hospital, Tianjin 300052, China.; 5 Department of Pulmonary and Critical Care Medicine, Shanghai General Hospital, Shanghai Jiao Tong University School of Medicine, Shanghai 201620, China.; 6 Department of Pulmonary and Critical Care Medicine, Zhongshan Hospital, Fudan University, Shanghai 200032, China.; 7 State Key Laboratory of Experimental Hematology, Key Laboratory of Immune Microenvironment and Disease (Ministry of Education), Department of Immunology, School of Basic Medical Sciences, Tianjin Medical University, Tianjin 300070, China.

**Keywords:** sepsis, multifunctional nanotherapeutics, peptide-based nanomicelles, anti-microbial nanodevice, anti-inflammation

## Abstract

Multifunctional nanodevices that simultaneously destruct bacteria and control detrimental inflammation are anticipated to serve as an effective therapy for sepsis. Toll-like receptor 2 (TLR2) and TLR4 signaling pathways are pivotal to the pathogenesis of sepsis from the clinical data analysis. Herein, inspired by understanding of the molecular interactions between TLR2/4 and their natural ligands, we *de novo* design an amphiphilic, helical, cationic peptide R18, which potently inhibits the activation of both TLR2 and TLR4, and eradicates bacteria. Such inhibition is primarily achieved by binding of R18 to TLR2 or to both TLR4 ligand and receptor, which interferes with the ligand-receptor interactions. We also define the essential role of the hydrophobic and cationic amino acid residues in the peptide sequence in these multi-actions. By conjugating R18 to the self-assembled PEGylated phospholipid-based nanomicelles (designated as M-CR18), the antibacterial activity and the stability are significantly enhanced. The mechanistic studies reveal that M-CR18 effectively eliminates bacteria through triple-destruction on bacterial membrane integrity, biofilm formation, and bacterial flagellar assembly when compared with the molecular R18. The* in vivo* efficacy of M-CR18 is validated in infectious mouse models of cecal ligation and puncture as well as *Pseudomonas aeruginosa*-induced acute lung injury, and a non-infectious mouse model of lipopolysaccharide (LPS)-induced pulmonary inflammation. Finally, M-CR18 can effectively eliminate clinically present drug-resistant bacteria. This study provides a *de novo* design principle for multifunctional nanodevices with immunomodulatory and antibacterial activities, which represent a novel class of nano-antibiotics for the treatment of bacterial infection-mediated pneumonia and sepsis.

## 1. Introduction

Sepsis is a life-threatening medical condition that occurs when the immune system overreacts toward bacterial infection, resulting in overwhelming systemic inflammation and multiple organ dysfunction 1, 2. Currently, it causes about 11 million deaths per year globally, including neonatal sepsis 3. The rise of aging population and the emergence of antibiotic-resistant bacteria pose new challenges in preventing and treating sepsis. To date, there is still no effective pharmacological treatment available in the clinics to treat sepsis except for antibiotics and the cornerstone supportive care for organ dysfunction (i.e., fluid resuscitation), both of which sometimes make patients more vulnerable to nosocomial infections in the late phase of sepsis 4. Therefore, it is urgent to develop novel therapeutic strategies to combat this notorious disease.

The overwhelming inflammation at the acute phase of sepsis is believed to be the key stage affecting the mortality of the septic patients 5. Specifically, when pathogens invade the host, the phagocytic cells, such as macrophages, can sense and internalize these microorganisms and launch innate immune reaction to eradicate them while alarming the adaptive immune system 6. This host defense process is effective and controllable in most cases, but at certain circumstances, it may lose control and cause enormous production of pro-inflammatory cytokines including tumor necrosis factor-α (TNF-α) and interferon-γ (IFN-γ), which in turn induces inflammatory cell death (i.e., PANoptosis) in septic patients 7, 8. Such pathogen triggered uncontrolled host inflammatory reactions (i.e., cytokine storm) ultimately lead to the dysfunction and paralysis of the immune system. Therefore, an effective treatment at the early phase of sepsis is critical, and a multitasking strategy that can potently kill bacteria and simultaneously regulate the excessive inflammatory responses is required to effectively treat this complex disease.

Toll-like receptor signaling pathways play a critical role in the initiation of cytokine storm in sepsis. Among the ten identified human Toll-like receptors (TLRs), TLR4 and TLR2 are the two major contributors to the pathogenesis of sepsis 9, 10, as they sense Gram-negative and Gram-positive bacteria, respectively, to launch inflammatory reactions. It has been found that TLR4 knockout mice are resistant to Gram-negative bacteria-induced septic shock 11, and TLR2-deficient mice have increased survival rates compared to wild-type mice in a polymicrobial sepsis model 12. Furthermore, the blockade of TLR2 or TLR4 signaling by antagonistic antibodies or small molecule inhibitors successfully decreases disease severity in sepsis mouse models 13-15. Thus, multifunctional therapeutic agents that simultaneously inhibit both TLR2 and TLR4 activation while killing bacteria may serve as a promising strategy to combat sepsis. However, it is challenging to have a potent agent capable of achieving such a task.

Bioactive peptides are functional biopolymers that can be *de novo* designed or derived from nature with immunoregulatory and antimicrobial activities. For example, naturally existed antimicrobial peptides (AMPs) are key players in the host-defense mechanism against the invasion of various pathogens including bacteria, fungi, viruses and parasites 16, 17, through the disruption of microorganism membranes. This working mechanism makes AMPs more advantageous over traditional antibiotics due to less chance for the development of drug resistance. In addition to elimination of microbes, some AMPs have been found to be able to reduce harmful inflammation. For instance, the human host-defense peptide cathelicidin, LL-37, is capable of modulating TLR4-triggered inflammatory responses via binding to the TLR4 agonist lipopolysaccharides (LPS) to block the ligand-receptor interaction and downstream signaling 18, 19. Thus, the natural multifunctional AMPs could serve as a model for *de novo* designing functionality-directed synthetic AMPs that can simutaneously regulate TLR4/2 signaling and eradicate bacteria to combat sepsis.

In this study, we *de novo* designed a unique amphiphilic cationic helical peptide, R18, in order to achieve a dual-action on bacterial killing and TLR inhibition. R18 was designed based on the molecular docking analysis on TLR2/4 interacting with their agonists and by utilizing the helical wheel projection template. Such a design enabled R18 to interefere with the ligand-receptor interactions of TLR2 and TLR4, leading to inhibition of their downstream signaling pathways. In addition, R18 was capable of eradicating bacteria. The effect of the peptide sequence on this novel dual activity was further investigated by amino acid mutations in both hydrophobic and cationic regions of R18. To improve the solubility of R18 in the physiological condition and enhance its antibacterial activities, the self-assembled PEGylated phospholipid-based nanomicelles were applied to fabricate the R18-conjugated nanomicelles as M-CR18. It was found that, M-CR18 was superior to R18 in disrupting biofilm formation and bacterial membrane integrity, resulting in a stronger bacterial killing activity. Further transcriptomic analysis revealed that M-CR18 significantly down-regulated the bacterial genes responsible for the flagellar assembly, which caused the decrease in bacterial motility. Finally, the *in vivo* efficacy of M-CR18 was evaluated in infectious and non-infectious acute lung injury (ALI) mouse models. The antimicrobial effects of M-CR18 were also examined on clinically present drug-resistant bacterial species. This study provided a *de novo* design principle to construct multifunctional peptide with immunomodulatory and antibacterial acitivities. The nanoform of the peptide represented a novel generation of nano-antibiotics for the treatment of bacterial infection-mediated pneumonia and sepsis.

## 2. Results

### 2.1. The importance of TLR2/4 in sepsis and the design principle of a dual-functioned peptide for TLR2/4 inhibition and bacteria eradication

**Figure 1:**
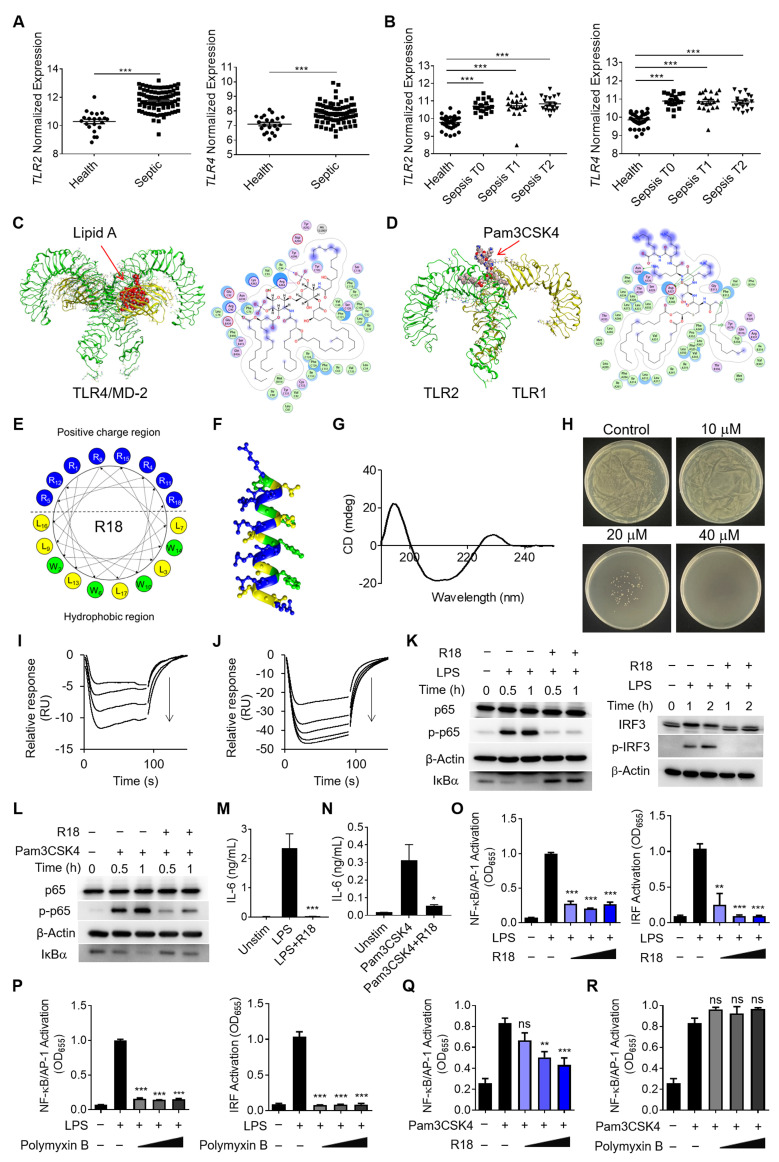
** The *de novo* design of the dual-function peptide R18 with bacterial killing ability and potent inhibitory activity on both TLR2 and TLR4 signaling pathways.** (A, B) The gene expressions of *TLR2* (left) and *TLR4* (right) in pediatric sepsis patients (A) and adult sepsis patients at different admission stages (T0: newly admitted, T1: 1 h of admission, T2: 3 h of admission) (B) in comparison with healthy controls. (C, D) The 3-D ribbon structures (left) and the binding pockets (right) showing the molecular interaction of lipid A with the TLR4/MD-2 complex (C) and Pam3CSK4 with the TLR1/2 heterodimer (D); green dotted lines indicated hydrogen bonding. (E) The design and arrangement of amino acids in the peptide sequence of R18 based on the helical wheel projection template. (F) The predicted α-helix structure of R18; the blue, yellow and green color encoded different amino acid residues of R, L and W, respectively. (G) The circular dichroism (CD) spectrum of R18 in PBS. (H) The plate colony analysis demonstrating the antibacterial activity of R18 at different concentrations on *E. coli*. (I, J) Negative SPR signals of R18 injected onto the TLR4/MD2-immobilized CM5 chip (I) and TLR2-immobilized CM5 chip (J); R18 concentrations: 0.039, 0.078, 0.156 and 0.313 μM for TLR4/MD2 and 0.039, 0.078, 0.156, 0.313 and 0.625 μM for TLR2; arrows indicated the direction of increasing concentration. (K) The immunoblots showing the inhibition of LPS-induced phosphorylation of p65 (p-p65) and degradation of IκBα for NF-κB activation (left) as well as the phosphorylation of IRF3 (p-IRF3) for IRF activation (right) by R18 in THP-1 cell-derived macrophages; β-Actin as the internal control; R18 = 5 μM. (L) Inhibition of Pam3CSK4-induced phosphorylation of p65 (p-p65) and degradation of IκBα in THP-1 cell-derived macrophages; β-Actin as the internal control; R18 = 5 μM, (M, N) The effects of R18 on the production of the pro-inflammatory cytokine IL-6 upon LPS (M) or Pam3CSK4 (N) stimulation for 24 h in THP-1 cell-derived macrophages; R18 = 10 μM. (O, P) The inhibition of LPS-induced NF-κB/AP-1 (left) and IRF (right) activation by R18 (O) and polymyxin B (P) in THP-1 reporter cell-derived macrophages; R18 and polymyxin B = 2.5, 5 and 10 μM. (Q, R) The inhibition of Pam3CSK4-induced NF-κB/AP-1 activation by R18 (Q) and polymyxin B (R) in THP-1 reporter cell-derived macrophages; R18 and polymyxin B = 2.5, 5 and 10 μM. LPS = 10 ng/mL, Pam3CSK4 = 10 ng/mL; N = 3. The data is presented as the mean ± SEM. ns: not significant, *p < 0.05, **p < 0.01, ***p < 0.001 vs. the stimulation group unless otherwise indicated.

As TLR2 and TLR4 are the main pattern recognition receptors for sensing the Gram-negative and Gram-positive bacteria, respectively, to trigger inflammatory reactions, their activation is expected to contribute significantly to the pathogenesis of sepsis 9, 10. To confirm this, we analyzed the GEO database of pediatric and adult septic patient samples for the correlation of TLR2 and TLR4 expressions with the disease development and progression in sepsis (**Figure S1A and B**). It was found that the pediatric septic patients had significantly higher expressions of *TLR2* and *TLR4* in the whole blood samples than the healthy controls (**Figure 1A**); the expression of the inflammatory gene *IL1B* was positively correlated with that of *TLR2* and *TLR4* in the pediatric septic patients (**Figure S1C and D**). Similarly, the analysis of the GEO database of the adult septic patients revealed that *TLR2* and *TLR4* expressions were elevated regardless the disease stages of the patients when compared with the healthy controls (**Figure 1B**), and were positively correlated with *IL1B* expression (**Figure S1E and F**). These findings provided strong evidences of TLR2/4 inhibition as a promising therapeutic strategy to reduce detrimental inflammation for treating both pediatric and adult septic patients.

In order to convey such a strategy into therapeutics, one could design potent TLR2/4 antagonists by understanding the molecular interaction between the receptors and their ligands. LPS and the synthetic Pam3CSK4 are the known prototypical ligands for TLR4 homodimers and TLR1/2 heterodimers, respectively 20, 21; particularly, the lipid A component of LPS is the main moiety that interacts with the glycoprotein MD-2 for TLR4 binding for the immunostimulatory activity of LPS. Thus, the lipid A and Pam3CSK4 were employed as the effective motifs to perform the molecular docking of these ligands with their specific receptors. As shown in **Figure 1C**, lipid A was fitted into the MD-2 pocket of a TLR4/MD-2 complex by hydrophobic interactions, hydrogen bonds and ionic bonds, where the five lipid chains of lipid A exhibited a favorable steric complementarity inside the large hydrophobic pocket of MD-2. For Pam3CSK4 and TLR1/2 interaction, the molecular docking analysis revealed that two lipid chains of Pam3CSK4 were inserted into the hydrophobic pocket of TLR2 while one lipid chain positioning in the TLR1 hydrophobic pocket by two π-H interactions (**Figure 1D**). These analyses suggested the critical role of the hydrophobic interactions in assisting the binding of TLR4 and TLR2 with their ligands.

Based on the above molecular docking analysis, we aimed to develop a unique dual-function peptide that is capable of interacting with both TLR2 and TLR4 to block their ligand-receptor binding for activation and killing bacteria. The peptide was *de novo* designed to have an amphiphilic helical structure where the hydrophobic region can insert into the hydrophobic pocket of TLR2 and TLR4, and the cationic region helps kill bacteria. Using the helical wheel projection method, the R18 peptide containing 18 amino acids in the sequence (RWLRRWLRLWRRLWRLLR-NH_2_) was constructed (**Figure 1E**), in which 8 arginine (R) residues were placed on one side of the helical structure (**Figure 1F**) forming a cationic region, and 4 tryptophan (W) and 6 leucine (L) residues were positioned on the other side to form a hydrophobic region. To assess if R18 can form the α-helix structure as expected, the circular dichroism (CD) spectroscopy was conducted. It was found that R18 had a high portion of α-helix (**Figure 1G**), which was consistent with the software prediction (**Table S1**). These results confirmed our helical structural design principle in constructing R18.

Next, we evaluated the dual-function of R18. The antibacterial activity of R18 was examined on cultured *Escherichia coli*. It was found that R18 was able to completely inhibit the bacterial growth in a solid agar plate at a concentration of 40 μM (**Figure 1H**). Then, we assessed whether R18 could directly bind to the TLR4/MD-2 complex and TLR2 using the surface plasmon resonance (SPR) technique. Interestingly, peptide dose-dependent negative SPR signal profiles were observed with the addition of R18 to the immobilized TLR4/MD-2 or TLR2 (**Figure 1I and J**); such an unusual phenomenon may be due to the conformational changes of the individual peptides and/or the receptors upon interaction with each other as documented in the literature 22-24. Nevertheless, this result indicated the presence of specific interactions of R18 with the TLR4/MD-2 complex and TLR2. It was expected that these interactions would disrupt the binding of the ligands to TLR4 and TLR2, thereby inhibiting their downstream signal transduction. The immunoblotting results confirmed that R18 treatment reduced the TLR2- and TLR4-mediated activation of the transcription factors nuclear factor κB (NF-κB) and interferon regulatory factor (IRF) in THP-1 cell-derived macrophages (**Figure 1K and L**), where the phosphorylation of the NF-κB subunit p65 (p-p65) and IRF3 (p-IRF3) as well as the degradation of the NF-κB inhibitor factor IκBα were reduced by R18. Moreover, R18 significantly down-regulated the production of the pro-inflammatory cytokines interleukin-6 (IL-6), monocyte chemoattractant protein-1 (MCP-1) and TNF-α under LPS or Pam3CSK4 stimulation (**Figure 1M and N**; **Figure S2**). These results demonstrated that R18 was capable of inhibiting both TLR2- and TLR4-mediated inflammatory responses by blocking the ligand-receptor interactions while having the antibacterial activity.

Polymyxin B (PMB), a cationic cyclic peptide, is one of the last-line antibiotics to treat drug-resistant bacterial infection in the clinic. Studies have shown that polymyxin B is also a TLR4 antagonist by neutralizing the bacterial product LPS 25. To assess the potency of our newly designed R18, we made the head-to-head comparison between R18 and polymyxin B on their TLR4 and TLR2 inhibitory activities. Using THP-1 reporter cell-derived macrophages, we found that both R18 and polymyxin B were able to effectively inhibit LPS-induced activation of the transcription factors NF-κB/activator protein-1 (AP-1) and IRF at different peptide concentrations (**Figure 1O and P**). In addition, R18 could also potently inhibit Pam3CSK4-induced NF-κB/AP-1 activation of TLR2 signaling in a peptide concentration-dependent manner (**Figure 1Q**); however, polymyxin B had no effect on TLR2 inhibition (**Figure 1R**). It should be noted that the TLR inhibitory activity of R18 was specific to TLR2 and TLR4, but not to other endosomal TLRs, such as TLR3 and TLR7/8 (**Figure S3**). These observations suggested that R18 was superior to the clinical used polymyxin B in regulating both TLR2 and TLR4-associated inflammatory cascades, which are often up-regulated in the polymicrobial sepsis.

Next, we further determined whether R18 could bind to LPS like PMB for TLR4 inhibition. Using the fluorescence polarization technique, we demonstrated that R18 was able to bind to LPS (**Figure S4A**) with a Kd value of 2.23 ± 1.03 μM. However, R18 could hardly bind to the TLR2 ligand Pam3CSK4 with a Kd value of 663.57 ± 112.26 μM (**Figure S4B**). Such a large Kd value indicated low affinity of R18 to Pam3CSK4. Furthermore, it was found that R18 did not bind to the other TLR2 ligand LTA, where the mP value did not change with increasing concentrations of LTA (no concentration-dependent effect) (**Figure S4C**).

To examine whether the specific binding of R18 to TLR2 and TLR4 contributed to the observed immune modulation function, we performed the TLR inhibition tests using a special R18 pre-treatment and wash experimental procedure. As shown in **Figure S5A**, THP-1 reporter cell-derived macrophages were pretreated with R18 for 12 h, followed by PBS washing twice to remove the weakly bound R18 on the cells and the free R18 in the medium prior to the TLR ligand stimulation. Interestingly, with such a pre-treatment and wash procedure, it was found that R18 could still inhibit the LPS-induced activation of NF-κB/AP-1 and IRF (**Figure S5B**), but the inhibitory effect was lower than that of co-treatment experiment (R18 co-incubation with LPS) shown in **Figure 1O**. These results suggested that R18 inhibited TLR4 pathway through both neutralizing LPS and binding to TLR4. Differently for TLR2 inhibition, the pre-treatment of R18 had similar inhibitory effect on TLR2 activation trigger by Pam3CSK4 and LTA ligands when compared with the co-treatment experiments (**Figure 1Q** and **Figure S5C-E**), suggesting that R18 inhibited TLR2 predominantly through binding to the receptor.

### 2.2. Effects of the amino acid sequence of R18 on the TLR inhibitory and antibacterial activities

**Figure 2:**
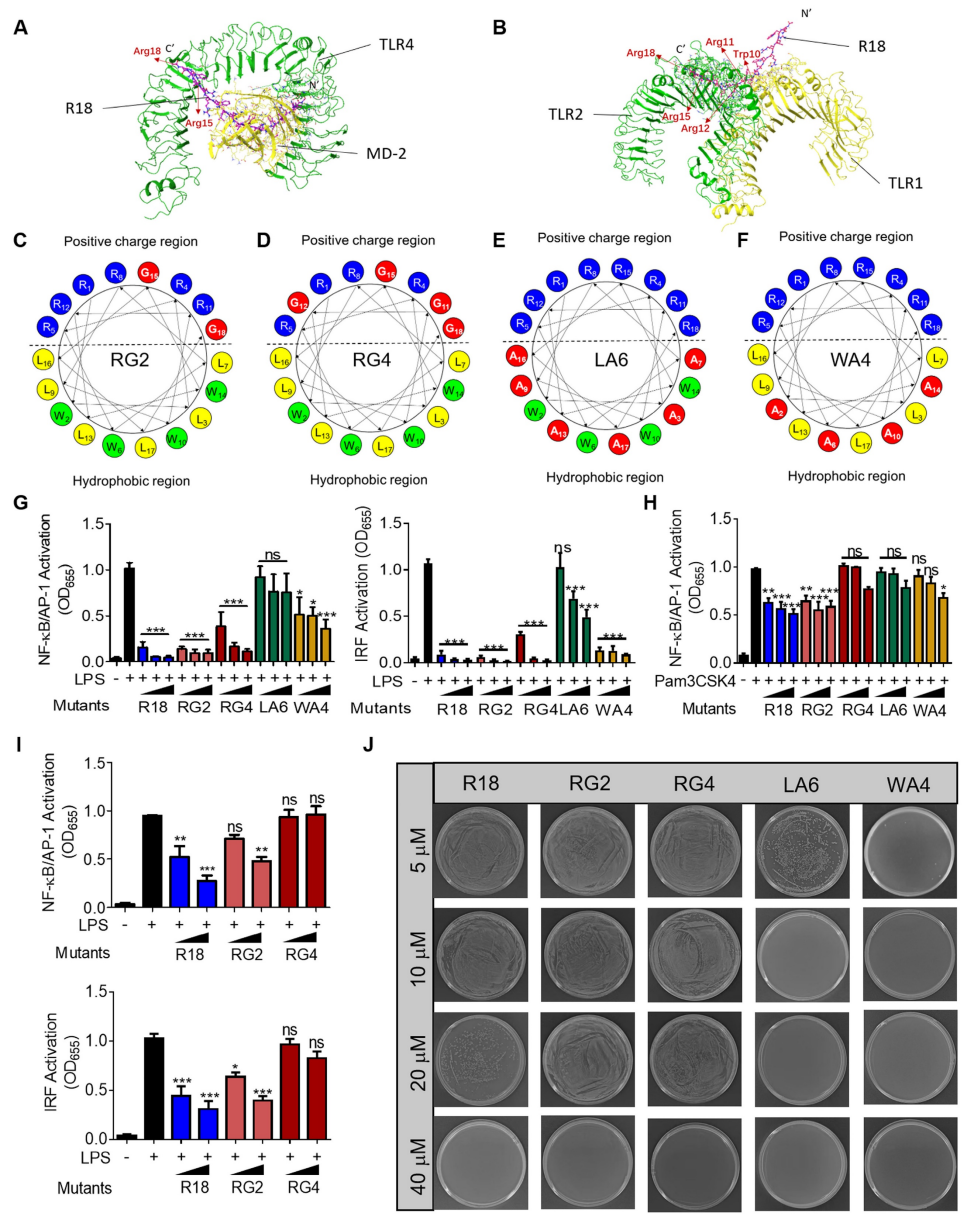
** Effects of amino acid sequence of R18 on the TLR inhibitory and antimicrobial activities.** (A, B) Molecular docking analysis showing the binding of R18 to the TLR4/MD-2 complex (A) and the TLR1/2 heterodimer (B); the essential R residues of R18 for the TLR binding were labeled by pink color. (C, D) The helical wheel projections showing the mutations of the R residues of R18 to glycine (G) as RG2 (C) or RG4 (D) based on the molecular docking results on TLR4/MD-2 or TLR1/2, respectively; red color indicated the mutated residues. (E, F) The helical wheel projections demonstrating the mutations of the hydrophobic residues L and W of R18 to alanine (A) as LA6 (E) and WA4 (F), respectively; red color indicated the mutated residues. (G) The inhibition of TLR4-mediated NF-κB/AP-1 (left) and IRF (right) activation by the four mutant peptides (RG2, RG4, LA6 and WA4) in comparison with R18 in THP-1 reporter cell-derived macrophages; LPS = 10 ng/mL, peptide concentration = 1.25, 2.5 and 5 μM. (H) The inhibition of TLR2-mediated NF-κB/AP-1 activation by the four mutant peptides compared with R18 in THP-1 reporter cell-derived macrophages; Pam3CSK4 = 10 ng/mL, peptide concentration = 1.25, 2.5 and 5 μM. (I) The inhibition of LPS-induced NF-κB/AP-1 (top) and IRF (bottom) activation by RG2 and RG4 compared with R18 at lower peptide concentrations (0.3125 and 0.625 μM) in THP-1 reporter cell-derived macrophages; LPS = 10 ng/mL. (J) Representative pictures showing the colony formation of *E. coli* treated with the four mutant peptides compared with R18 at different peptide concentrations. The data is presented as the mean ± SEM. ns: not significant, *p < 0.05, **p < 0.01, ***p < 0.001 vs. the stimulation group unless otherwise indicated.

In order to better understand how the amino acid residues in R18 contribute to its novel dual function of TLR inhibition and bacterial killing, we first conducted molecular docking analysis to view the molecular interaction of R18 with the TLR4/MD2 complex and the TLR1/2 complex. The ribbon diagrams of the 3-D structures revealed that the 15th and 18th arginine of R18 interacted with TLR4 (**Figure 2A**), and the arginines at position 11, 12, 15 and 18 as well as the 10th tryptophan of R18 were associated with the TLR1/2 complex (**Figure 2B**). The binding free energy (∆G_binding_) of the TLR4/2 specific ligands or R18 to TLR4 and TLR2 were determined using the MM-GBSA method. It was found that R18 bound to the receptors with an estimated free binding energy of -36.12 kcal/mol for TLR4/MD-2 and -237.76 kcal/mol for TLR1/2, which are much lower than those of specific ligands lipid A and Pam3CSK4 to the receptors, suggesting that R18 binds to TLR2/4 stronger than their specific ligands (**Table S2**). Based on the above prediction, we constructed four R18 mutants with the following amino acid alterations: (i) both the 15th and 18th arginine residues were replaced with glycine (G) as RG2 (**Figure 2C**); (ii) the four arginine residues at positions 11, 12, 15 and 18 were mutated to G as RG4 (**Figure 2D**); (iii) the six leucine (L) residues and (iv) the four tryptophan (W) residues were replaced with less hydrophobic and smaller alanine (A) as LA6 and WA4, respectively (**Figure 2E and F**). The inhibitory activity of these mutated peptides on TLR4 and TLR2 signaling were evaluated on the THP-1 reporter cell-derived macrophages in comparison with that of R18. It was found that the ability of LA6 and WA4 on TLR4 inhibition was significantly decreased (**Figure 2G**), and that on TLR2 inhibition was even diminished (**Figure 2H**).

In contrast, the inhibitory activity of RG2 on both TLR4 and TLR2 signaling remained unaffected while that of RG4 was mildly altered on TLR4 signaling and abolished on TLR2 signaling (**Figure 2G and H**). These results suggested that the hydrophobic residues L and W were essential for the inhibitory activity of R18 on TLR4 and TLR2. However, the effects of cationic residue R of R18 on the TLR inhibitory ability depended on the position and numbers of mutated R; this could be clearly seen at lower peptide concentrations, where RG4 had no effects on the inhibition of LPS-induced NF-κB and IRF activation (**Figure 2I**), indicating R residues at positions 11, 12, 15 and 18 were important for the TLR inhibitory activity of R18.

The effects of these amino acid mutations on the antibacterial activity of R18 were next examined. It was found that RG2 and RG4 had a similar activity on bacterial killing in comparison with R18 (**Figure 2J**). Surprisingly, WA4 and LA6 exhibited a stronger antibacterial activity than R18 (**Figure 2J**; **Figure S6**). This indicated that low hydrophobicity in WA4 and LA6 may be beneficial for bacterial killing, whereas mutations in 2 or 4 arginine residues did not alter such an activity. Collectively, R18 appeared to be the optimal design with an effective dual-function of both TLR inhibition and bacterial elimination.

### 2.3. Fabrication of R18-decorated lipid-core nanomicelles

**Figure 3:**
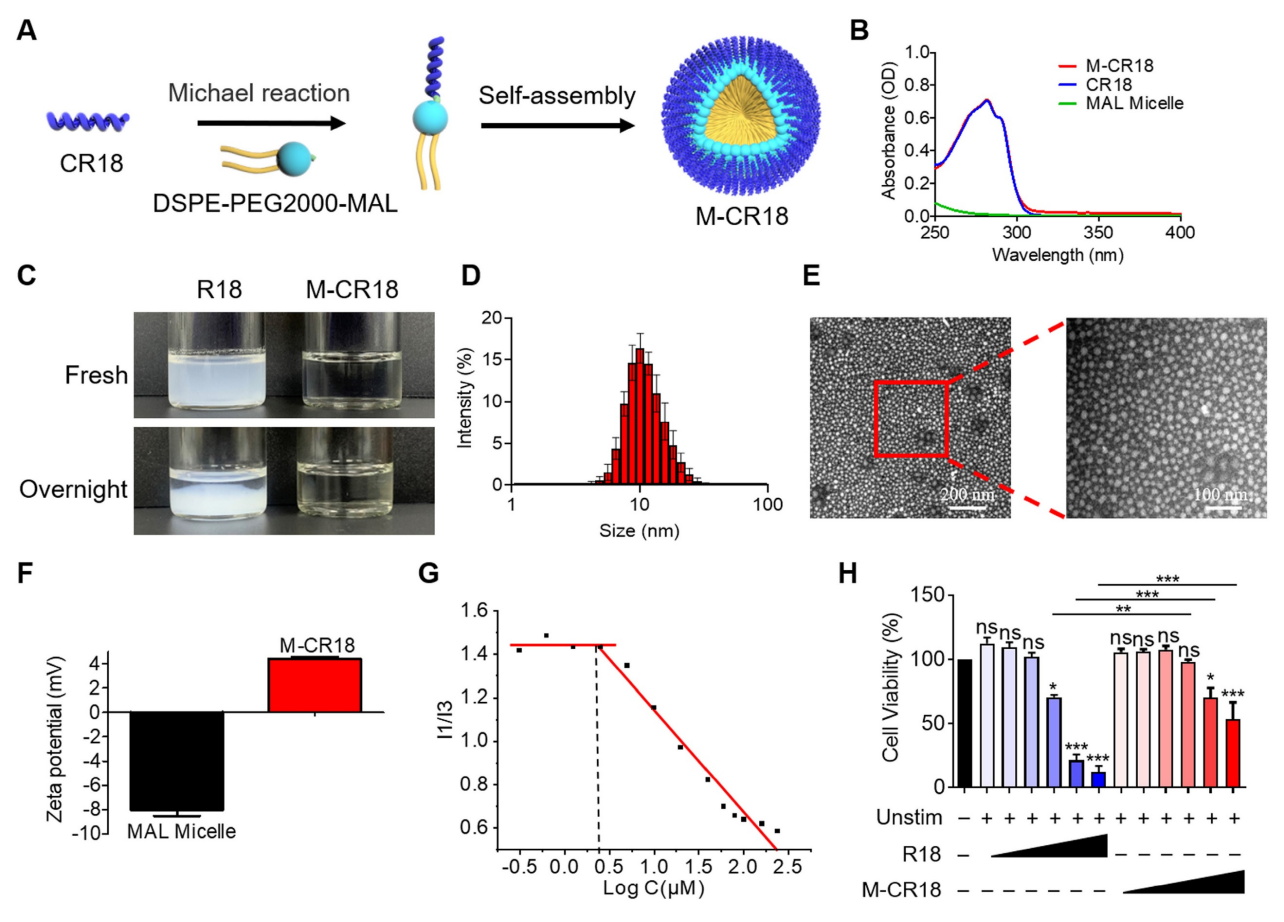
** Fabrication and characterization of the R18-decorated lipid-core nanomicelles M-CR18.** (A) A scheme showing the conjugation of CR18 to the DSPE-PEG2000 phospholipid and the assembly into the nanomicelles M-CR18. (B) The UV-vis spectra of M-CR18, R18 and the unmodified nanomicelles (MAL micelles). (C) Photographs of R18 and M-CR18 right after preparation and after overnight storage. (D) The hydrodynamic diameter distribution of M-CR18 by DLS. (E) The representative TEM images of M-CR18; the red dotted square indicated the location of the zoom-in image on the right. (F) Zeta potentials of M-CR18 and the unmodified MAL micelles. (G) The intensity ratio of the first (I1) to the third (I3) peak of pyrene fluorescence as a function of the M-CR18 concentrations (i.e., peptide concentrations) to determine the CMC of M-CR18 at the intersection of the two fitted straight lines. (H) The cell viability of THP-1 cell-derived macrophages treated with R18 and M-CR18 at different concentrations for 24 h; M-CR18 and R18 = 2.5, 5, 10, 20, 40 and 50 μM. The data is presented as the mean ± SEM. ns: not significant, *p < 0.05, **p < 0.01, ***p < 0.001 vs. the unstimulated control unless otherwise indicated.

Although R18 displayed desired dual functionality of bacterial killing and TLR inhibition, its low aqueous solubility may hinder the in vivo application of R18. To overcome this problem, we conjugated R18 onto the phospholipid-based nanomicelles to i) improve the solubility, ii) reduce the cytotoxicity, and iii) enhance the antibacterial activity of R18. This was achieved by introducing a cysteine (C) residue at the N-terminal of R18, named CR18, which can react with a PEGylated phospholipid, 1,2-distearoyl-sn-glycero-3-phosphoethanolamine-poly(ethylene glycol)2000-maleimide (DSPE-PEG2000-MAL) through Michael addition to form the R18-conjugated lipid amphiphiles; these amphiphiles then self-assembled into the R18-decorated lipid-core nanomicelles designated as M-CR18 (**Figure 3A**). The R18 conjugation to the nanomicelles was confirmed by the UV-visible spectra, where both M-CR18 and R18 had an overlapping absorption spectrum with a peak at 280 nm, but the unmodified nanomicelles did not have any absorption at 280-300 nm (**Figure 3B**). The conjugation percentage of CR18 on the nanomicelles was estimated to be 85.90 ± 8.79%.

As expected, by conjugating CR18 to the lipid-core nanomicelles, the stability and dispersibility of the formed M-CR18 was significantly better than R18 in PBS. R18 itself tended to form milky colloidal suspensions and precipitate overnight (**Figure 3C**), while M-CR18 formed clear solution and remained stable overnight. Dynamic light scattering (DLS) analysis showed that M-CR18 had an intensity-based hydrodynamic diameter of 10.96 ± 1.48 nm (**Figure 3D**); under the transmission electron microscopy (TEM), M-CR18 displayed a relatively uniform and spherical morphology (**Figure 3E**), with an average diameter of 12.23 ± 3.83 nm (**Figure S7**), comparable with the hydrodynamic diameter obtained by DLS. Because the unmodified MAL micelles carry negative charges with a zeta potential around -8 mV (**Figure 3F**), these negative charges could neutralize the positive charges of R18 on M-CR18, resulting in a zeta potential of approximately 4 mV for M-CR18 (**Figure 3F**). The stability of M-CR18 was also reflected from its relatively low critical micelle concentration (CMC) of 2.53 ± 0.27 μM measured by the pyrene fluorescence analysis based on the changes in the fluorescence intensity ratios of the first (373 nm) to the third peak (384 nm) (I1/I3) as a function of M-CR18 concentraitons (i.e. peptide concentrations) (**Figure 3G and Figure S8**). More importantly, the cytotoxicity of M-CR18 to the THP-1 cell-derived macrophages (**Figure 3H**) as well as the endothelial cell line Eahy-926 (**Figure S9**) was significantly reduced when compared with R18. These results demonstrated that M-CR18 nanomicelles had better dispersibility, higher stability and lower cytotoxicity than R18 peptides, and hence were desirable for *in vivo* applications.

### 2.4. M-CR18 exhibited enhanced antibacterial activity with comparable anti-inflammatory ability *in vitro* when compared with R18

**Figure 4:**
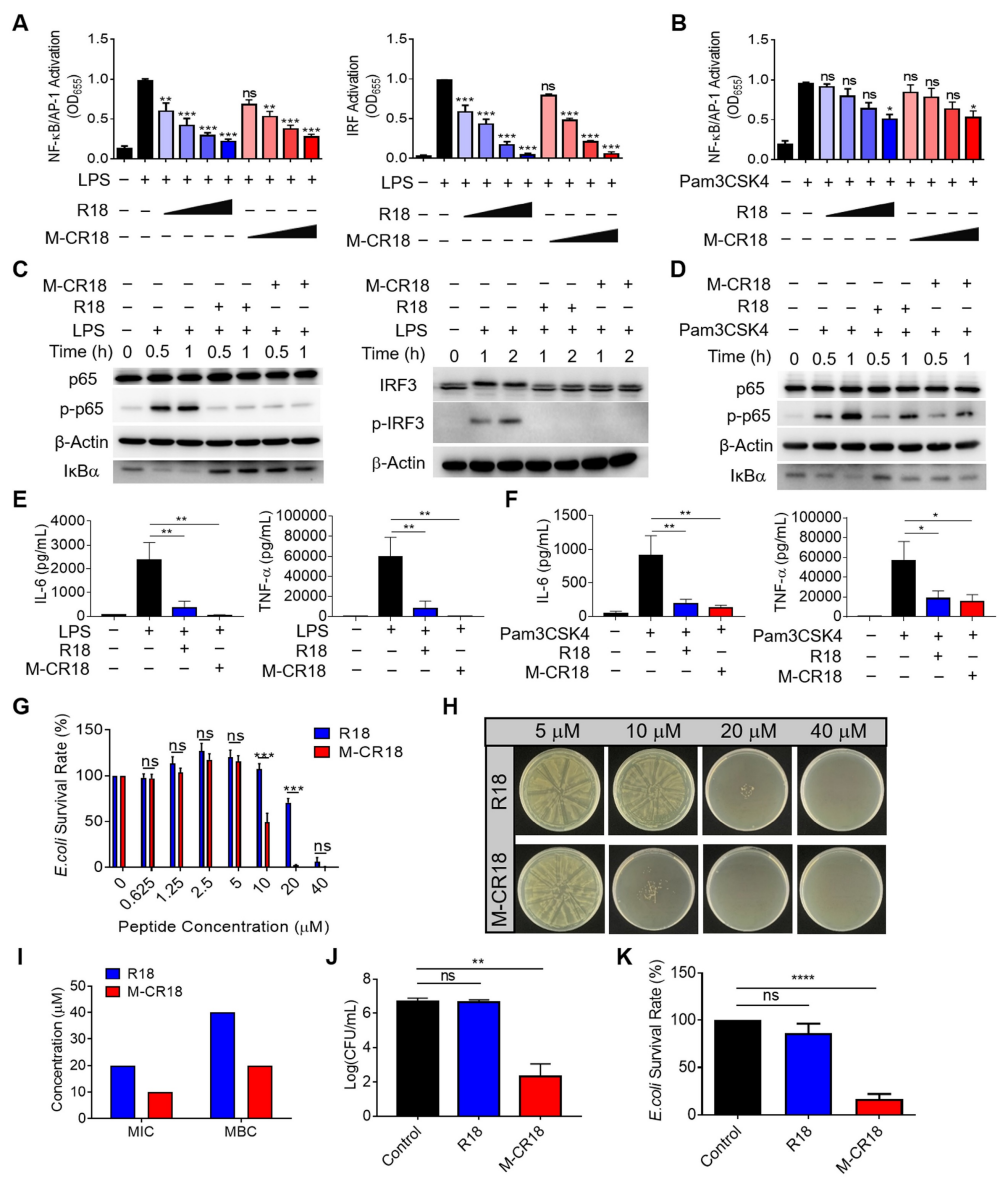
** The anti-inflammatory and antibacterial activities of M-CR18 *in vitro*.** (A, B) The inhibition of TLR4-mediated NF-κB/AP-1 (left) and IRF (right) activation (A) and TLR2-mediated NF-κB/AP-1 activation (B) by M-CR18 and R18 in THP-1 reporter cell-derived macrophages; R18 and M-CR18 = 0.625, 1.25, 2.5 and 5 μM. (C) The immunoblots showing the inhibition of LPS-induced NF-κB activation (p-p65 and IκBα degradation) (left) and IRF3 activation (p-IRF3) (right) by R18 and M-CR18 in THP-1 cell-derived macrophages; β-Actin as the internal control; R18 and M-CR18 = 5 μM. (D) The immunoblots showing the inhibition of Pam3CSK4-stimulated NF-κB activation (p-p65 and IκBα degradation) by R18 and M-CR18 in THP-1 cell-derived macrophages; β-Actin as the internal control; R18 and M-CR18 = 5 μM. (E, F) The effects of R18 and M-CR18 on the production of IL-6 (left) and TNF-α (right) upon LPS (E) and Pam3CSK4 (F) stimulation for 24 h in THP-1 cell-derived macrophages. (G) The effects of M-CR18 on the survival rate of *E. coli* compared with R18. (H) The effects of M-CR18 on the plate colony formation of *E. coli* in comparison with R18. (I) The estimated MIC and MBC of M-CR18 and R18 against *E. coli*. (J) The number of colonies of *E. coli* treated with the same concentration of M-CR18 and R18 for 20 h; M-CR18 and R18 = 10 μM. (K) The survival rate of *E. coli* treated with the same concentration of M-CR18 and R18 for 20 h; M-CR18 and R18 = 10 μM. N ≥ 3; LPS = 10 ng/mL, Pam3CSK4 = 10 ng/mL. The data is presented as the mean ± SEM. ns: not significant, *p < 0.05, **p < 0.01, ***p < 0.001, ****p < 0.0001 vs. the stimulation group unless otherwise indicated.

Since M-CR18 displayed better physiochemical characteristics than R18, we next evaluated how this nano-conversion affected their anti-inflammatory and antibacterial activities when compared with the R18 peptides. It was found that M-CR18 had equal ablity as R18 on inhibiting both TLR4 and TLR2 signaling pathways (**Figure 4A and B**), but did not have any effect on TLR3, TLR7/8, and other inflammatory cytokine signaling pathways including IFN-β, TNF-α and IL-1α (**Figure S10**). Such an inhibitory activity on TLR4 and TLR2 signaling was also confirmed by immunoblotting, with the decrease in the phosphorylation of p65 and IRF3, and IκBα degradation (**Figure 4C and D**). Furthermore, M-CR18 was able to effectively down-regulate the production of pro-inflammatory cytokines IL-6, TNF-α and IL-1β under LPS or Pam3CSK4 stimulation similar to R18 (**Figure 4E and F**; **Figure S11**). These results demonostrated that R18 conjugation to the lipid nanomiclles imparted M-CR18 the potent TLR inhibitory and anti-inflammatory activities comparable with R18. It should be noted that similar to R18, M-CR18 was able to bind to LPS, but its binding ability was lower than that of R18, possibly due to steric hindrance effects resulting from the micelle formation (**Figure S12**). In addition, M-CR18 displayed the same inhibitory mechanisms as R18 on TLR2 and TLR4 through interfering with the ligand-receptor interactions (**Figure S5**).

In addition to TLR inhibition, M-CR18 nanomicelles surprisingly exhibited a stronger antibacterial acitivity than R18. As shown in **Figure 4G**, while both R18 and M-CR18 were able to inhibit bacterial growth in a peptide concentration-dependent manner, M-CR18 displayed much greater bacterial killing ability at concentrations of 10 and 20 μM. The minimum bactericidal concentration (MBC) of M-CR18 and R18 was estimated by observing colony-forming units (CFUs) on Luria Broth agar plates using micro-double dilution method (**Figure 4H**), whereas the minimum inhibitory concentration (MIC) was determined by analyzing the optical density (OD) at 600 nm of the bacterial suspensions for the bacterial survival rate. It was found that both MIC and MBC of M-CR18 were lower than those of R18 (**Figure 4I**). To better compare the antibacterial ability of M-CR18 with that of R18, the same concentration (10 μM) of M-CR18 (MIC) and R18 (half of MIC) was used to treat *E. coli*; we found that M-CR18 significantly reduced the bacterial colony formation (**Figure 4J**) and suppressed the bacterial survival rate (**Figure 4K**) but R18 did not. These results indicated that converting the R18 peptide into a nanodevice (M-CR18) could significantly enhance the antibacterial potency while maintaining the same TLR inhibitory effectiveness.

### 2.5. Multiple antibacterial effects of M-CR18

**Figure 5:**
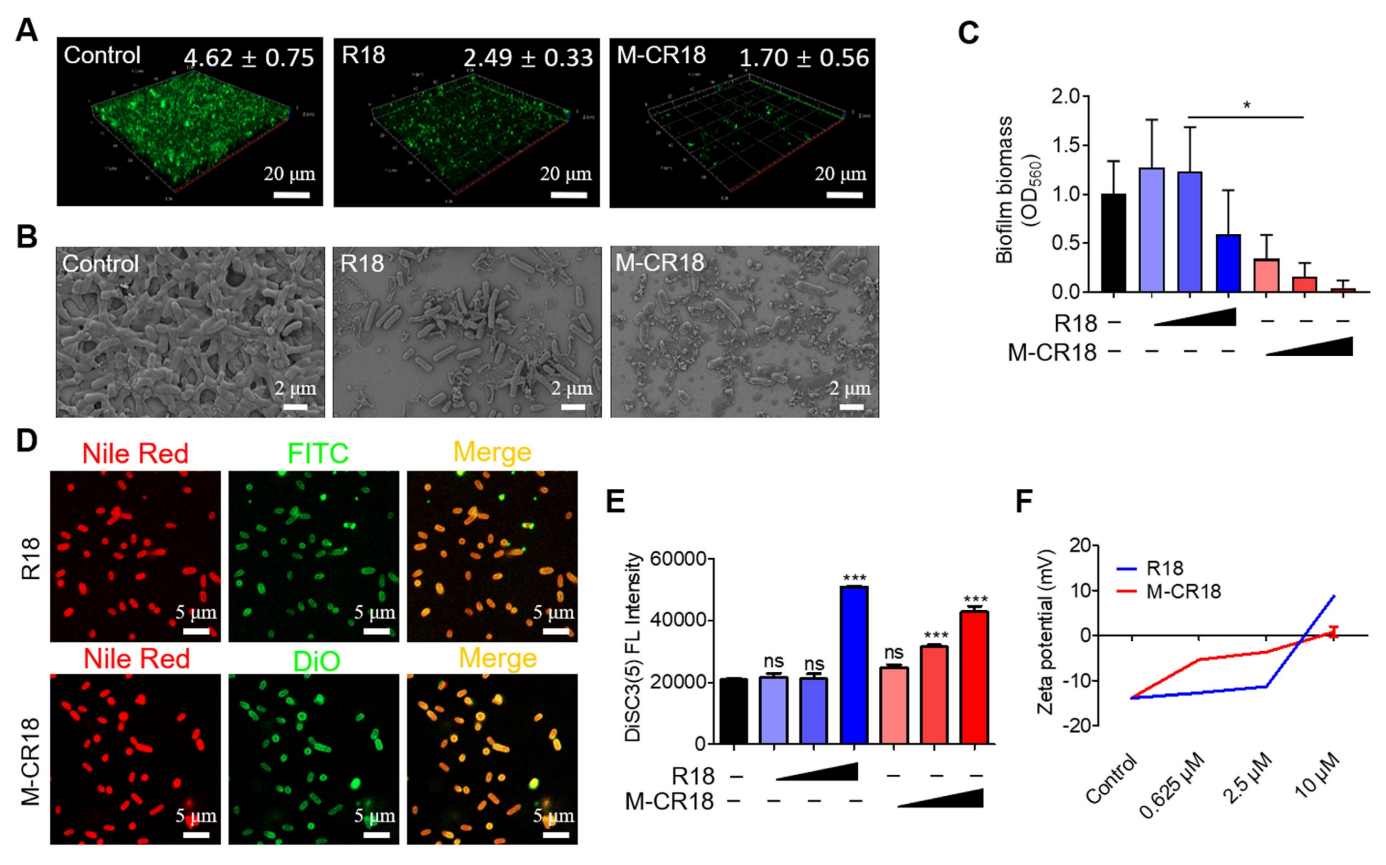
**The multiple actions of M-CR18 on eliminating bacteria *in vitro*.** (A) The confocal microscopic images of calcein-stained bacterial biofilms of *E. coli* upon the treatments of M-CR18 and R18; M-CR18 and R18 = 10 μM. The average biofilm thickness (μm) was indicated on the image. (B) The SEM images of the *E. coli* biofilms treated with M-CR18 or R18; M-CR18 and R18 = 10 μM. (C) The inhibitory effects of M-CR18 and R18 on *E. coli* biofilm formation by crystal violet staining assay; M-CR18 and R18 = 0.625, 2.5 and 10 μM. (D) The fluorescence images showing the affinity of FITC-labeled R18 (green) and DiO-labeled M-CR18 (green) with *E. coli* stained with Nile red on the bacterial membranes (red); FITC-R18 = 20 μM, DiO-M-CR18 = 10 μM; treatment time = 4 h. (E) The disruption of the inner membrane integrity of *E. coli* probed by DiSC3(5) fluorescence upon the treatment of M-CR18 or R18 at different concentrations for 2.5 h; M-CR18 and R18 = 0.625, 2.5 and 10 μM. (F) Zeta potential of the bacteria *E. coli* treated with different concentrations of M-CR18 or R18 for 2.5 h. The data is presented as the mean ± SEM. ns: not significant, *p < 0.05, ***p < 0.001 vs. the untreated control unless otherwise indicated.

As M-CR18 exhibited superior antibacterial activity to R18, we were wondering how such bactericidal effects were achieved. It is known that biofilm formation is necessary for bacterial growth, and it increases the tolerance to the antimicrobial agents. Thus, we first examined the effect of M-CR18 on the bacterial biofilm formation via calcein-staining. It was found that the green fluorescence signals of the biofilm were significantly decreased by R18 and were almost gone with the M-CR18 treatment (**Figure 5A**). The same phenomenon was observed by scanning electron microscopy (SEM), where both the thickness of the biofilm and the number of bacteria were largely reduced by R18 or M-CR18, and such effects were more dramatic (with few intact bacteria left) with M-CR18 treatment (**Figure 5B**). Quantitative analysis with crystal violet staining further confirmed these results (**Figure 5C**).

We next speculated whether R18 and M-CR18 could act on modulating the bacterial membrane structure and properties as reported by most discovered antimicrobial peptides 26. To test our speculation, the bacterial membranes were stained with Nile red (red), and the bacteria were treated with FITC-labeled R18 (green) or DiO-labeled M-CR18 (green) to observe their affinity to the bacterial membranes by the confocal microscopy. The fluorescence images showed that the red and green signals were co-localized, indicating the interaction of R18 and M-CR18 with the bacterial membranes (**Figure 5D**). We then investigated whether this interaction would disrupt the bacterial inner membrane integrity by using a membrane potential sensitive probe, DiSC3(5) 27. This cationic fluorescent probe is at the self-quenched state when accumulating in the intact bacterial inner membrane, and becomes fluorescent when the membrane is disrupted to release the probe. Through this approach, we found that both M-CR18 and R18 at a higher concentration could increase the fluorescence intensity of DiSC3(5); however, M-CR18 was more potent to do so at a lower concentration, suggesting that M-CR18 had greater ability in disrupting the inner membrane integrity than R18 (**Figure 5E**). This enhanced bacterial membrane disruption effect of M-CR18 may be explained by its stronger capability in altering the bacterial membrane potential, as the zeta potentials of M-CR18 treated bacteria were higher than those of R18 treated ones at the concentration below 2.5 μM (**Figure 5F**). Collectively, these results suggested that despite both R18 and M-CR18 displayed multiple antibacterial actions, the nanodevice M-CR18 was more potent than the molecular R18.

### 2.6. Defining the mechanism(s) for the enhanced antibacterial activities of M-CR18

**Figure 6:**
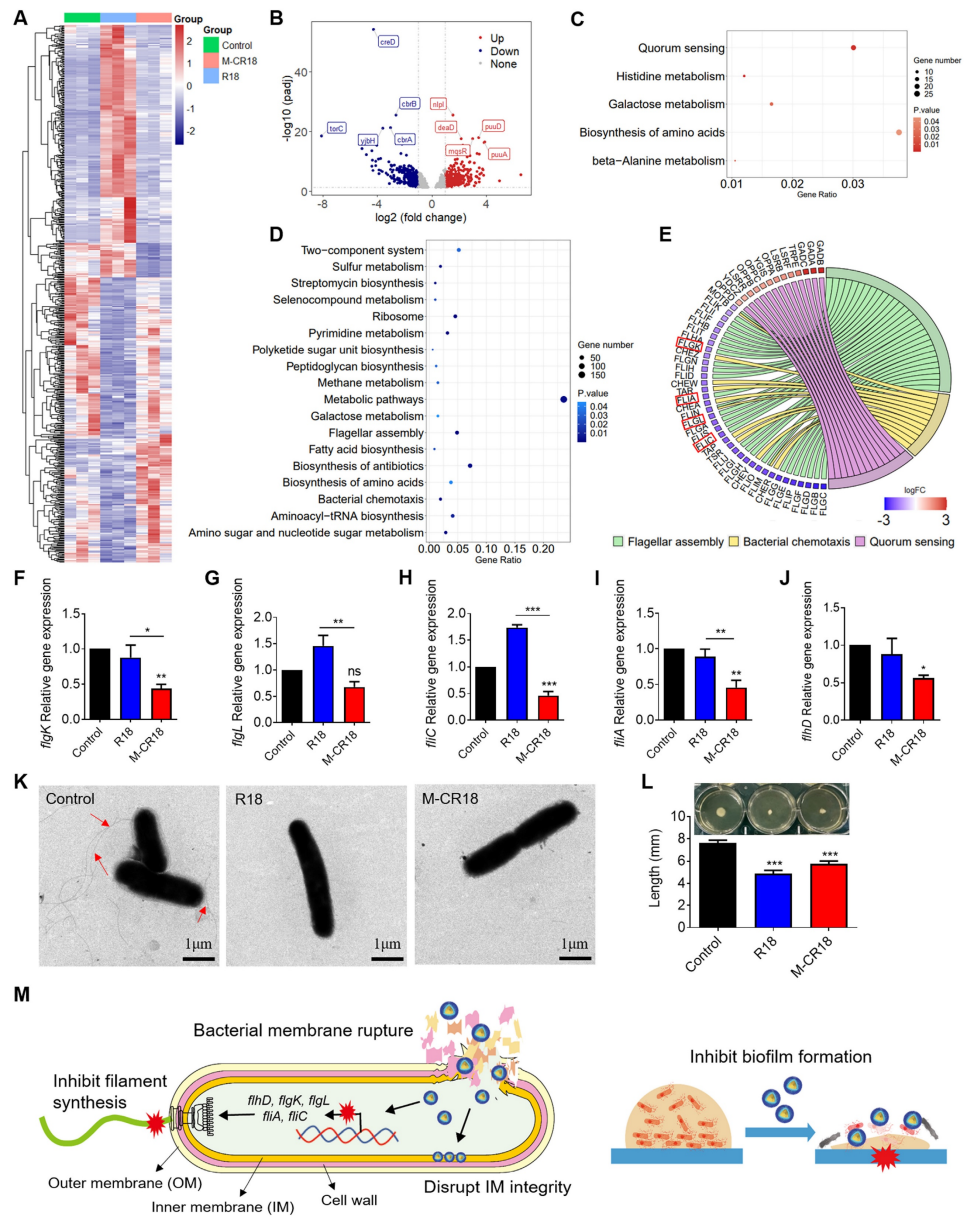
** The transcriptome analysis of the antibacterial effects of M-CR18 on *E. coli*.** (A) The heat map of differentially expressed genes (DEGs) in *E. coli* affected by R18 or M-CR18 treatment; padj < 0.05, log|FC| > 1. (B) The volcano plot of the up-regulated (red) and down-regulated (blue) genes by M-CR18 compared with R18 with the criteria of padj < 0.05 and log|FC | > 1. (C, D) The bubble map showing the enriched KEGG up-regulation (C) and down-regulation (D) pathways by M-CR18 compared with R18 with p value < 0.05. (E) The enrichment analysis showing the circular plot of DEGs by M-CR18 compared with R18 related to the bacterial motility; red: up-regulated DEGs, blue: down-regulated DEGs. (F-J) The expressions of flagellar assembly genes *flgK* (F), *flgL* (G),* fliC* (H), *fliA* (I) and *flhD* (J) upon the R18 and M-CR18 treatments by qRT-PCR. (K) The bacterial flagellum formation visualized by TEM; R18 and M-CR18 = 80 μM; scale bar = 1 μm. (L) The quantitative analysis of the flagellum-dependent bacterial motility based on the colony diffusion in the 0.5% agar; the representative images shown on the top and the analysis of the largest diffusion distance across the colony shown at the bottom. (M) A schematic diagram demonstrating the proposed antibacterial mechanisms of action for M-CR18. The data is presented as the mean ± SEM. ns: not significant, *p < 0.05, **p < 0.01, ***p < 0.001 vs. the untreated control unless otherwise indicated.

To identify the possible molecular mechanism(s) for the enhanced antibacterial effects of M-CR18, the prokaryotic transcriptome analysis was performed on R18- and M-CR18-treated bacteria to compare the similarities and differences between the two treatments. The heatmap revealed that the differentially expressed genes affected by R18 or M-CR18 in *E. coli* had different patterns, suggesting that the effects of M-CR18 and R18 were not entirely identical. (**Figure 6A**). The volcano plot presented 357 up-regulated genes and 316 down-regulated genes for M-CR18 compared with R18 (**Figure 6B**). GO analysis results showed that the up-regulated genes by M-CR18 compared with R18 were mainly enriched in amino-acid betaine and amine metabolic process (**Figure S13A**). However, the down-regulated genes were associated with bacterial motility, particularly motility dependent on cilium or flagella (**Figure S13B**). By performing KEGG pathway enrichment analysis of differentially expressed genes, we found that the up-regulated genes by M-CR18 compared with R18 were mainly enriched in the quorum sensing system, histidine metabolism, galactose metabolism, amino acid biosynthesis, and β-alanine metabolism pathways (**Figure 6C**). On the other hand, the down-regulated genes were enriched in pathways related to bacterial motility including flagellar assembly and bacterial chemotaxis, and in metabolic pathways such as pyrimidine metabolism, amino and nucleotide sugar metabolism, sulfur metabolism, antibiotic biosynthesis, and aminoacyl tRNA biosynthesis (**Figure 6D**).

We further focused on the down-regulated pathways associated with the bacterial motility, and found that M-CR18 significantly decreased many genes related to flagellar assembly process compared with R18 (**Figure 6E**). These genes included *flgK* and *flgL* encoding hook-associated proteins (**Figure 6F and G**), *fliC* encoding filamentous proteins (**Figure 6H**), and *fliA* controlling the expression of late flagellum-related genes (**Figure 6I**). In addition, *flhD* that governs the upstream transcriptional activators for the flagellar synthesis was also significantly down-regulated by M-CR18 (**Figure 6J**), suggesting that M-CR18 could effectively inhibit flagellar synthesis in *E. coli*. Such an inhibitory effect was confirmed by TEM, where the untreated *E. coli* had elongated flagellar structures (red arrows) while the number of bacterial flagella was significantly reduced with R18 or M-CR18 treatment (**Figure 6K**; **Figure S14**). Moreover, the bacterial flagellum-dependent motility was assessed on 0.5% agar plates, and the results showed that both R18 and M-CR18 treatments were able to reduce the swarming movement of bacteria (**Figure 6L**). The reduced bacterial motility was also found in M-CR18 treated *P. aeruginosa* (PA103) (**Figure S15**). It is worth noting that the expressions of *CheY*, *CheA*, *CheZ* and *CheR* genes that govern the chemotactic signal transduction for bacterial movement were all decreased by M-CR18 compared with R18 as well (**Figure 6E**), which may also contribute to the enhanced antibacterial activity of M-CR18.

In summary, M-CR18 exhibited enhanced antibacterial activities through the following acting mechanisms: i) disruption of the bacterial inner membrane integrity, ii) cessation of flagellar synthesis, and iii) inhibition of biofilm formation. Together, these actions allowed M-CR18 to effectively rupture the the bacterial membrane to cause bacterial death, inhibit bacterial motility to stop invasion, adhesion and aggregation, and decrease the bacterial biofilm formation for bacterial growth and survival (**Figure 6M**).

### 2.7. The anti-inflammatory and antibacterial efficacy of M-CR18 in non-infectious and infectious mouse models

**Figure 7:**
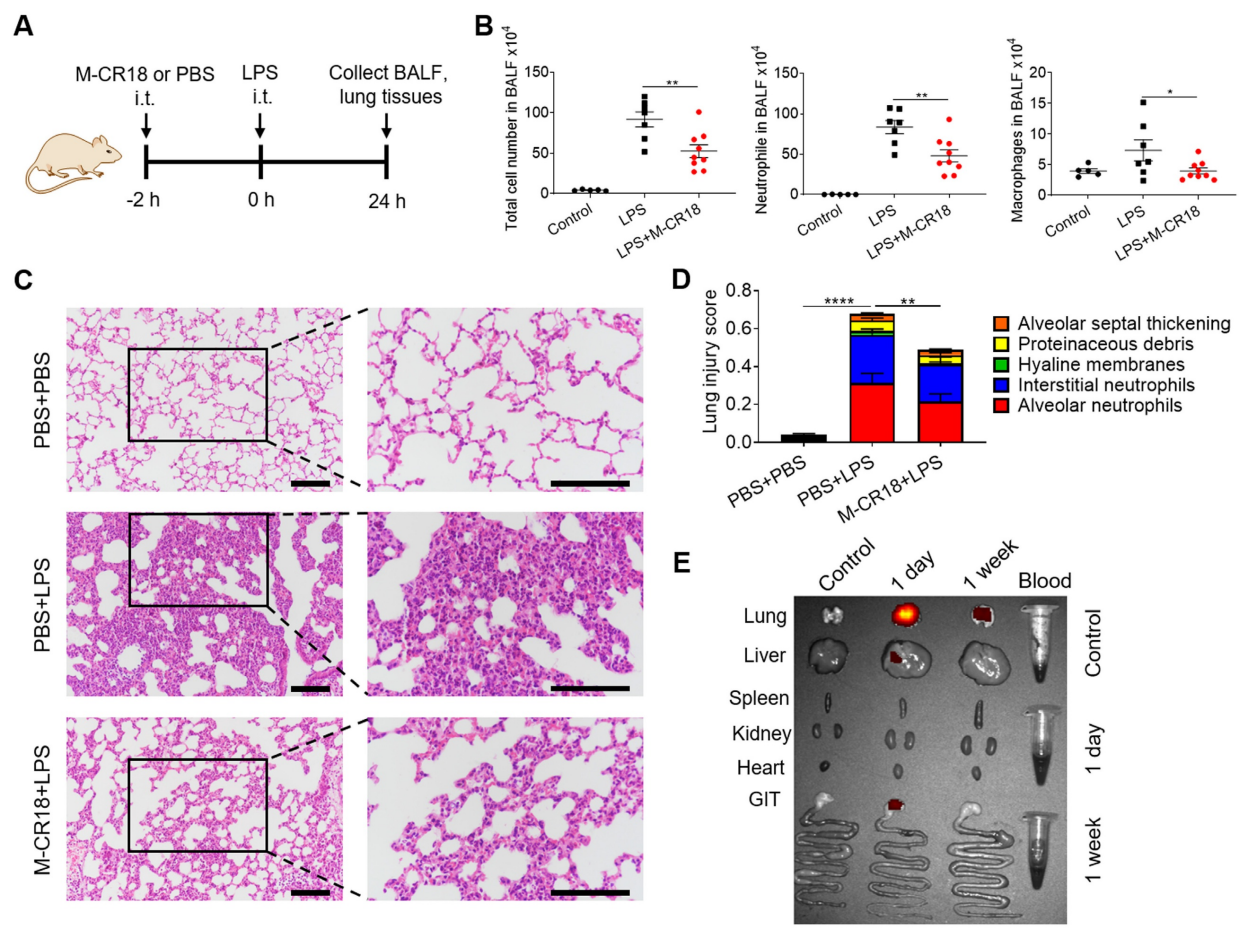
** Anti-inflammatory effects of M-CR18 in the LPS-induced ALI mouse model.** (A) A schematic diagram showing the LPS-induced ALI mouse model; M-CR18 (30 nmol/kg) was administered intratracheally 2 h before LPS (10 mg/kg) challenge through the same route for 24 h. (B) The total number of cells (left), neutrophils (middle) and macrophages (right) in the BALF. (C) The representative histological images of lung sections stained with H&E; scale bar = 50 μm. (D) The lung injury score analyzed from (C) with five pathological features; N = 5-9. (E) The distribution of M-CR18 in different organs/tissues in ALI mice 1 day and 1 week after the administration (i.t.) of DiR-labeled M-CR18 (30 nmol/kg); N = 3. The data is presented as the mean ± SEM. *p < 0.05, **p < 0.01, ****p < 0.0001.

The anti-inflammatory effects of M-CR18 *in vivo* were first evaluated on a non-infectious, LPS-induced ALI mouse model. M-CR18 was administered (30 nmol/kg) intratracheally (i.t.) 2 h before LPS challenge (10 mg/kg) through the same route for 24 h (**Figure 7A**). At the end of the model, the bronchoalveolar lavage fluid (BALF) and lung tissues were collected for the analysis of lung inflammation and injury. It was found that the M-CR18 treatment significantly reduced the number of total cells, neutrophils and macrophages in the BALF of LPS-induced ALI mice (**Figure 7B**). The histopathological analysis of the lungs revealed that M-CR18 effectively decreased LPS-induced lung inflammation and injury (**Figure 7C**), where the lung injury score was quantified with five pathological features including alveolar neutrophils, interstitial neutrophils, hyaline membranes, alveolar protein exudation, and alveolar septal thickening (**Figure 7D**; **Figure S16**). These results demonstrated that M-CR18 exhibited potent anti-inflammatory activity against non-infectious LPS-induced ALI.

To investigate the biodistribution profile of M-CR18 in mice upon intratracheal instillation, the major organs/tissues of lungs, liver, spleen, kidneys, heart, gastrointestinal track (GIT) and blood were harvested one day or one week after the administration of DiR-labeled M-CR18. The ex vivo fluorescence images of the organs/tissues showed that M-CR18 was primarily accumulated in the lung one day after M-CR18 administration, with minor accumulation in the liver; the fluorescence signals were still observed in the lung with much less intensity one week after the administration (**Figure 7E**). These results suggested that M-CR18 had a preferential distribution in the lung through i.t. administration and could be excreted out overtime.

**Figure 8:**
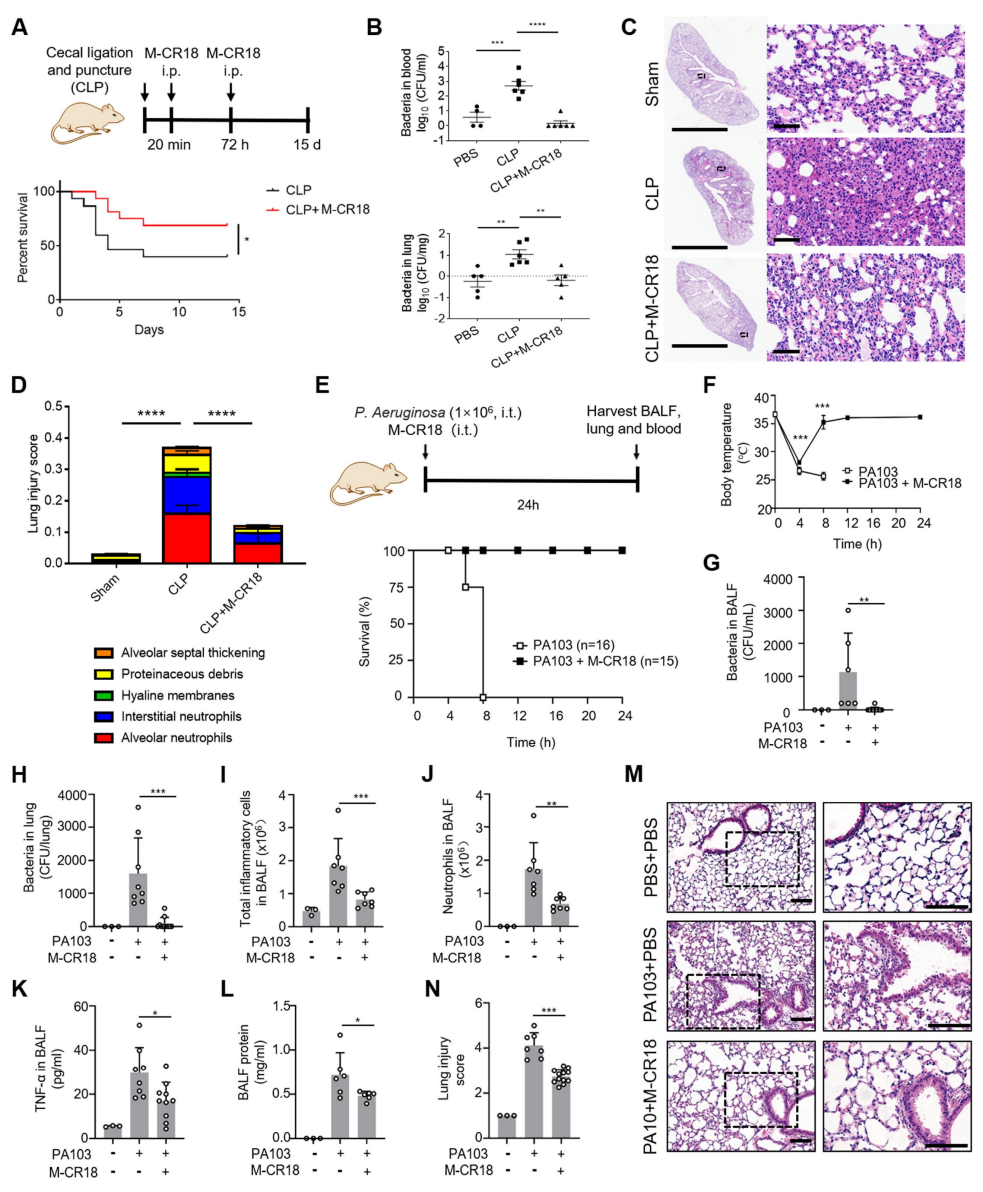
** The protective effects of M-CR18 in CLP-induced sepsis and *P. aeruginosa*-induced ALI mouse models.** (A) The effect of M-CR18 on the mouse survival rate (bottom, N = 15-16) in a CLP-induced sepsis mouse model (top); M-CR18 (120 nmol/kg) was given through i.p. injection at 20 min and 72 h post CLP procedure. (B) The bacterial load in the blood (top) and lungs (bottom) in mice 3 days after mild CLP induction with or without the M-CR18 treatment. (C) The representative histological images of the left lung with H&E staining 3 days after mild CLP induction; the zoom-in images of the black boxes shown on the right; scale bar = 4 mm (left) and 60 μm (right). (D) The lung injury score analyzed from the histological images in (C) with five pathological features; N = 4-6. (E) The effect of M-CR18 on the survival rate (bottom, N ≥ 15) in the *P. aeruginosa* (PA103)-infected (i.t.) ALI mouse model (top); M-CR18 = 120 nmol/kg, PA103 = 1×10^6^ CFU per mouse. (F) The changes of body temperature in mice infected with PA103 in lungs with/without M-CR18 treatment. (G, H) The bacterial load in the BALF (G) and the lung tissue (H) of PA103-infected mice. (I-L) The analysis of the number of the total cells (I) and neutrophils (J), TNF-α production (K) and total protein concentration (L) in the BALF of the PA103-infected mice with or without the M-CR18 treatment. (M, N) The histological images of lung sections stained with H&E (M) and the analyzed lung injury score (N) in the PA103-infected ALI mouse model; the zoom-in images of the black dotted boxes shown on the right; scale bar = 100 μm. The data is presented as the mean ± SEM. *p < 0.05, **p < 0.01, ***p < 0.001, ****p < 0.0001.

To evaluate both anti-inflammatory and antibacterial functions of M-CR18 *in vivo*, we adopted two infectious mouse models: the classical cecal ligation and puncture (CLP)-induced polymicrobial sepsis mouse model and the *P. aeruginosa* pulmonary infection mouse model. In the CLP-induced sepsis mouse model, M-CR18 (120 nmol/kg) was intraperitoneal (i.p.) injected 20 min and 72 h after CLP, and the mouse survival rate was recorded over a period of 15 days (**Figure 8A**). It was clearly seen that M-CR18 significantly increased the mouse survival rate, implicating its potent, dual anti-inflammatory and antibacterial ability. To better assess the dual-function of M-CR18 *in vivo*, a mild CLP-induced sepsis model was used. By examining the bacterial load in different organs/tissues, we found that M-CR18 treatment potently reduced the bacterial burden in the blood, lungs, heart, liver, spleen and kidneys during sepsis (**Figure 8B**; **Figure S17A-D**). The histopathological images revealed the reduced injuries in the lungs of the M-CR18 treated mice (**Figure 8C**). The lung injury score demonstrated that M-CR18 decreased all five pathological features during sepsis: infiltration of neutrophils in both alveolar and pulmonary interstitium, proteinaceous debris, alveolar septal thickening and hyaline membranes (**Figure 8D**; **Figure S17E-I**). In addition to the effects on the lung, M-CR18 significantly alleviated hepatocellular ballooning and reduced piecemeal necrosis of the liver. Moreover, M-CR18 effectively inhibited glomerular atrophy and tubular dilation with hemorrhage in the kidneys of mice under CLP (**Figure S17J**). These multiple lines of evidence suggested that M-CR18 was able to reduce the bacterial load, organ inflammation and injuries as well as the mortality rate during sepsis.

In the pulmonary infection mouse model, the M-CR18 was intratracheally administered together with the *P. aeruginosa* (PA103) challenge for 24 h, and the BALF and lung tissue were collected for analysis (**Figure 8E**). It was found that M-CR18 was able to 100% protect mice from PA103-induced death, whereas the untreated mice all died within 8 h after bacterial challenge (**Figure 8E**); the body temperature of PA103-infected mice returned to the normal level after 8 h with M-CR18 treatment (**Figure 8F**). The bacterial counts in the BALF and the lung of PA103-challenged mice were significantly lower in the M-CR18 group than in the untreated group (**Figure 8G and H**), confirming the potent antibacterial activity of M-CR18 in treating bacterial lung infection. Furthermore, M-CR18 treatment decreased the number of total infiltrated inflammatory cells (**Figure 8I**) and neutrophils (**Figure 8J**) as well as the pro-inflammatory cytokine TNF-α level (**Figure 8K**) and the protein concentration (**Figure 8L**) in the BALF. The histopathological analysis of the lung sections revealed that the lung inflammation and injury were significantly reduced by M-CR18 treatment (**Figure 8M and N**). All these results demonstrated that M-CR18 was capable of eradicating bacteria while decreasing lung inflammation and injury during *P. aeruginosa* infection, confirming its dual antibacterial and anti-inflammatory activity *in vivo*.

Next, we evaluated the *in vivo* biosafety profile of M-CR18. M-CR18 was injected intraperitoneally twice (20 min and 72 h), a regime similar to the survival test of the CLP model, and the lung, heart, liver, spleen, kidneys and blood were collected 15 days later to conduct the toxicity examination (**Figure S18A**). Compared with the PBS control, M-CR18 did not affect the tissue mass index (tissue mass per mouse weight) for all tested organs (**Figure S18B**). The hematological analysis of the mouse blood showed no difference in the levels of red blood cells (RBC), hemoglobin (Hb) and hematocrit (HCT) (**Figure S18C**), and the levels of white blood cells (WBC), the percentage of neutrophils (NE), lymphocytes (LY) and monocytes (MO) between the PBS control and M-CR18 (**Figure S18D**). Furthermore, the biochemical analysis of the blood revealed that the levels of total protein (TP), albumin (ALB) and globulin (GLOB) were not altered by M-CR18 (**Figure S18E**), nor were the ions of sodium (Na^+^), calcium (Ca^2+^), phosphate (Phos) and potassium (K^+^) (**Figure S18F**). Moreover, M-CR18 did not affect the kidney function indicated by the urea and creatine (CREA) levels (**Figure S18G**), as well as the levels of liver and pancreas enzymes in the blood, including alanine transaminase (ALT), aspartate transaminase (AST) and alkaline phosphatase (ALP) (**Figure S18H**). From the histological analysis, M-CR18 caused negligible inflammation in the lung, and no histological damage was observed in other organs (**Figure S18I**). These biosafety test results suggested that the i.p. administration of M-CR18 was relatively safe in healthy mice.

One big challenge in facing infectious diseases in the clinic is the global rising of antibiotic resistance, of which current treatments are very limited 28. Since M-CR18 exhibited great antibacterial activity *in vitro* and *in vivo* with multiple actions, we rationally speculated that M-CR18 may possess the ability on killing the antibiotic-resistant bacteria commonly seen in the clinics. By assessing the MIC and MBC values of M-CR18 on a variety of clinically isolated drug-resistant bacterial strains, including *E. coli*, *P. aeruginosa*, *Acinetobacter baumannii* and the Gram-positive *Staphylococcus aureus* (MRSA) (**Figure S19**), we found that M-CR18 was effective on eliminating these antibiotic-resistant strains. This suggested that our *de novo* designed M-CR18 may serve as new antibacterial nanodevices for antibiotic-resistant bacteria.

## 3. Discussion

Sepsis or bacterial pneumonia caused by multidrug-resistant Gram-negative bacteria and subsequent recurrent infections has become a serious health problem and global health emergency 29, 30. Abundant evidence demonstrates that both the pathogen burden and the dysregulated host immune responses are the detrimental factors in sepsis. TLR4 and TLR2 are identified as key receptors participating in pathogenic inflammatory responses under bacterial infection. The multifunctional antibacterial drugs that can inhibit both TLR4 and TLR2 signaling pathways to control excessive inflammation are anticipated to be promising therapeutics to treat sepsis. In this study, we first performed the bioinformatic analysis on the pediatric and adult septic patient datasets to demonstrate the clinical significance of TLR4 and TLR2 inhibition as a promising therapeutic approach in treating sepsis. Then, inspired by the molecular docking results of TLR4 and TLR2 with their specific ligands, we *de novo* designed a unique, multifunctional peptide R18 that consists of an amphiphilic α-helix structure with cationic residues on one side and hydrophobic residues on the other side. This structure enabled R18 to specifically disrupt the ligand-receptor interactions of TLR4 and TLR2 to inhibit the inflammatory responses while having the ability in killing bacteria. To further enhance the performance of R18, we conjugated R18 to the DSPE-PEG2000 self-assembled nanomicelles as the nanoform M-CR18. M-CR18 had improved stablity in PBS, and exhibited more potent antibacterial activity through destruction of bacterial membranes, inhibition of biofilm formation and suppression of bacterial motility (by down-regulating genes of bacterial flagellar synthesis and assembly) when compared with R18. More importantly, M-CR18 showed potent protective effects in CLP-induced sepsis, *P. aeruginosa* infection- and LPS-induced ALI mouse models, and was able to effectively eliminate several clinically present drug-resistant bacterial strains. This study provided a *de novo* design strategy for synthetic bioactive peptides with multifunctions on immunomodulation and bacterial killing, and presented a novel next generation of nano-antibiotics for the treatment of bacterial infection-mediated pneumonia and sepsis.

### 3.1. The pivotal role of simultaneous control of both TLR4 and TLR2 signaling pathways in sepsis

Among the TLR family members, TLR4 senses Gram-negative bacteria by interacting with LPS, whereas TLR2 recognizes lipoproteins/lipopeptides and glycolipids of Gram-positive bacteria. In addition to the recognition of the pathogen products, they can respond to the endogenous damage-associated molecular patterns (DAMPs) to further exacerbate the inflammatory responses 31, 32. Thus, the activation of both TLR2 and TLR4 is expected to play a critical role in the pathogenesis of sepsis.

Through bioinformatic analysis of the pediatric and adult sepsis datasets, we found that the gene expressions of *TLR2* and *TLR4* were significantly up-regulated in PBMC or blood samples of sepsis patients compared with the healthy controls (**Figure 1A and B**). The gene expressions of *TLR2* and *TLR4* in these patients were also correlated with that of the inflammatory cytokine *IL1B* (**Figure S1C-F**). These analyses together with reported literatures 33 indeed confirmed the TLR2 and TLR4 up-regulation as a strong pathogenic factor in sepsis patients. Moreover, studies have shown that TLR4 knockout mice were resistant to Gram-negative bacteria-induced septic shock 11 whereas TLR2-deficient mice had increased survival rates compared with the wild-type ones in a polymicrobial sepsis model 12. The pharmacological blockade of TLR2 and TLR4 signaling by antagonistic antibodies or small molecule inhibitors (e.g., TAK-242 for TLR4) successfully reduced the disease severity in bacteria-induced sepsis mouse models 13-15. These multiple lines of evidence indicate that simultaneous inhibition of TLR2 and TLR4 activation may serve as a promising therapeutic strategy for controlling overwhelming inflammation in sepsis.

### 3.2. Designing new antimicrobial peptides with multifunctions for controlling inflammation and eliminating bacteria in sepsis

Some of the natural antimicrobial peptides (AMPs) has been found to have additional anti-inflammatory function. For example, the cationic amphiphilic AMP, gcIFN-20, is capable of neutralizing LPS to down-regulate LPS-induced pro-inflammatory cytokine production and alleviate lung lesions in endotoxemic mice 34. The host defense AMP, LL-32, can also antagonize LPS to attenuate the endotoxin-induced inflammatory responses 25. The insect AMP, papiliocin, can directly bind to the TLR4/MD-2 complex in order to block LPS-TLR4 interactions, which inhibits downstream signaling 35. However, only a few natural AMPs have been reported to act on both TLR2 and TLR4 signaling pathways. For example, cathelicidins were found to suppress both TLR2 and TLR4 activation through binding to the ligands, but not to the receptors 19, 36. On the other hand, the keratin 6a-derived AMPs were found to inhibit both TLR2 and TLR4 activation through binding to the receptors 37. More versatile molecular agents and nano-devices need to be developed to better manage the overwhelming inflammation and bacterial infection for treating sepsis.

When compared with the natural AMPs, the synthetic AMPs have emerged as promising antimicrobial agents for their low cost of production, versatile design for tailored functionality and easiness of modification. Particularly, the *de novo* design of AMPs using a reasonable amino acid layout provides more flexibility to achieve desired functional features. In this study, we aimed to construct a novel, multifunctional peptide that can simutaneously control bacteria infection and inhibit both TLR2 and TLR4 pathways. Inspired by the molecular docking of these receptors with their specific agonists (**Figure 1C and D**), we *de novo* designed a unique amphiphilic helical peptide R18 with cationic residues (R) arranged on one side and hydrophobic residues (L and W) on the other side based on a helical wheel projection template (**Figure 1E-G**). Arginine (R) is the cationic amino acid commonly seen in most AMPs; leucine (L) and tryptophan (W) were selected for their hydrophobicity and cell membrane anchoring capability to empower the amphiphilic property and the membrane affinity of R18. With such a design, R18 exhibited a dual-action of TLR inhibition (**Figure 1K-O and Q**) and bacterial killing (**Figure 1H**), which is rarely seen in other synthetic helical AMPs. Specifically, R18 can interfere with the ligand-receptor binding to suppress both TLR2 and TLR4 signaling (**Figure 1I and J**). On the other hand, R18 can destruct the integrity of the bacterial membranes, which in turn elliminates the bacteria (**Figure 5D-F**). In addition, R18 can also inhibit the formation of bacterial biofilms (**Figure 5A-C**), bacterial flagellar assembly, and bacterial motility (**Figure 6K and L**), contributing to the antibacterial activity. These unique properties make R18 a novel synthetic multifunctional therapeutic agent for treating sepsis.

It should be noted that this dual-activity can be programed by the peptide sequence design. When the hydrophobic residues W and L were replaced with less hydrophobic alanine (A), the mutated peptides LA6 and WA4 showed reduced anti-inflammatory activity, but enhanced antibacterial ability (**Figure 2E-H and J**). On the other hand, replacing two or four R residues with glycine (G) did not significantly affect the antibacterial activity of R18, but decreased its ability on TLR inhibition (**Figure 2C, D and G-J**). This structure-activity relationship analysis provides guidance for future design of purpose-directed new dual-action helical synthetic AMPs.

### 3.3. The novel regulatory activity of M-CR18 on bacterial flagellar assembly and motility

The bacterial flagella are responsible for the motility of bacteria, participating in bacterial adhesion and chemotaxis. Drugs that target flagella to disrupt the bacterial motility are not prone to induce drug-resistance as opposed to antibiotics that mainly act on essential processes for bacterial growth and survival 38-40. Therefore, targeting bacterial flagella to inhibit bacterial movement and adhesion may represent a promising strategy for the design and development of next generation antibiotics.

We serendipitously discovered that M-CR18 was able to potently inhibit bacterial flagellar synthesis and assembly in comparison with R18, resulting in decreased flagellar formation and motility of *E. coli* (**Figure 6**). The bacterial flagellar synthesis is regulated by many genes, of which the *flhCD* manipulator is at the most up-stream level, governing the expression of a number of other flagellar genes 41. We found that M-CR18 significantly down-regulated the expressions of *flhCD* as well as many other flagellar assembly genes (e.g., *fliC*, *fliA*, *flgL* and *flgK*) (**Figure 6E-J**). Studies have shown that the inactivation of *fliC* does not affect the growth rate of *E. coli*, but significantly reduces the bacterial motility, biofilm formation and antibiotic tolerance 42. In addition, it has been found that flagellar motility plays a key role in the early stages of *E. coli* infection 43. Based on these facts, our findings open up an avenue of synthetic nano-AMPs with capability of regulating bacterial flagellar gene expressions and functions.

It is worth mentioning that M-CR18 also regulated a panel of genes responsible for the bacterial chemotaxis. For example, the two major receptors for chemotactic signal transduction pathway of *E. coli*, Tsr and Tar, were significantly down-regulated by M-CR18 (**Figure 6E**). Furthermore, the expressions of *CheY*, *CheA*, *CheZ* and *CheR* were all decreased by M-CR18, which govern the signaling from chemoreceptors to the flagellar motor (**Figure 6E**). These lines of evidence indicated that M-CR18 was able to inhibit the flagellar motor rotation switch, and consequently impaired the bacterial chemotactic ability. This novel function together with bacterial killing and the potent anti-inflammatory activity make M-CR18 a promising new generation of nano-based antibiotics for the treatment of bacterial infection-triggered sepsis.

### 3.4. The advantages of multifunctional nano-AMPs as new therapy for sepsis

The fast-increasing cases of sepsis or pneumonia caused by multidrug-resistant Gram-negative bacteria in the clinics have become a global health emergency 29, 30. Currently, the peptide-based polymyxin B is the last line of antibiotics to fight multidrug-resistant Gram-negative bacteria. Although polymyxin B is capable of inhibiting TLR4-mediated inflammatory responses by antagonizing LPS 44, it has neurotoxicity and often causes some serious kidney problems 45, 46. In addition, polymyxin B is not effective against Gram-positive bacterial infection and the associated inflammation. Therefore, there is an urgent need to design new effective antibiotics to complement current treatments for sepsis.

In order to improve the stability and antibacterial performance of AMPs, many attempts have been made to reshape the AMPs into nanoform through molecular self-assembly or conjugation. For instance, the human defensin-6 mimetic peptide is designed to self-assemble into nanofibril networks on the *Staphylococcus aureus* surfaces to wrap bacteria and stop their invasion 47. The conjugation of AMPs onto the gold nanoparticles can enhance the stability of the peptides against proteases and increase their therapeutic efficacy in vivo 48. The AMP-based micelles of the chimeric antibacterial lipopeptide and amphiphilic poly-(lactic-co-glycolic acid)-poly(ethylene glycol) polymers (PLGA-PEG) display improved stability against serum proteases with good therapeutic effects on *P. aeruginosa* lung infection in mice 49. These studies suggest that the nanoform of AMPs significantly improves the pharmacokinetic profile, the bioavailability and the half-life of AMPs *in vivo*, contributing to the superior stability and antimicrobial activity over the molecular AMPs.

We employed the lipid-core nanomicelles made of DSPE-PEG to serve as the nanoparticle core to fabricate the nanoform of R18 as M-CR18 (**Figure 3A**) by conjugating R18 onto the nanomicelle surface. The DSPE-PEG nanomicelles possess desired properties of nanosize, biodegradability, stability and biocompatibility as promising therapeutic carriers 50. In fact, the DSPE-PEG2000 has been approved in the clinical use to deliver the anticancer drug doxorubicin (i.e., Doxil, LipoDox and Thermodox) 51. Conjugating R18 onto nanomicelles as M-CR18 significantly improved the solubility of R18 and reduced its toxicity to human cells (**Figure 3C and H**); more importantly, the M-CR18 exhibited enhanced antibacterial activity with a triple-destruction mechanism through disrupting bacterial membane integrity, biofilm formation, and flagellar assembly **(Figure 4G-K**; **Figures 5 and 6**) while retaining the potent inhibitory activity on TLR2 and TLR4 signaling (**Figure 4A-F**). This dual-action of M-CR18 was confirmed in both non-infectious and infectious ALI mouse models (**Figures 7 and 8**). Moreover, M-CR18 displayed robust activity in killing various clinically present Gram-positive and Gram-negative antibiotic resistance bacteria (**Figure S19**). These superior activities of M-CR18 may result from the following aspests: first, the formation of nanostructures can concentrate the peptide into a small space to boost the antibacterial activity; second, the nanostructure can confine peptides within a supramolecular scaffold to reduce the effects of salts and proteases in the physiological environment; third, the nanoscale allows for specific cellular or organ/tissue distribution in the body to reduce unwanted systemic toxicity 52.

At present, most of the anti-inflammatory and antimicrobial nanodevices are fabricated for carrying various non-specific anti-inflammatory molecular agents and antibiotics or made by silver nanoparticles 53-55. Differently, our developed M-CR18 achieves the potent anti-inflammatory and antimicrobial dual function by conjugating a simple, *de novo* designed multifunctional AMP on the nanomicelles. The anti-inflammatory activity of M-CR18 is specific by targeting and suppressing TLR2/4 pathways. In addition, M-CR18 is also unique for its novel inhibitory activity on the bacterial flagellar assembly, which potentially avoids the antibacterial resistance problem. Overall, M-CR18 represents a unique class of multifunctional nano-therapeutics for treating sepsis and other bacterial infectious diseases.

## 4. Conclusions

We have designed and constructed a unique class of amphiphilic helical peptide-decorated nanomicelles that exhibit both potent immunomodulatory and antibacterial activities. Intruged by the clinical data analysis on the correlation between TLR2/4 expressions and the prognosis of the septic patients, and the molecular docking on the ligand-receptor interation of TLR2/4, we *de novo* designed the amphiphilic cationic peptide R18 with an α-helix structure. R18 could potently inhibit both TLR2 and TLR4 signaling while having antibacterial activity. It was found that the inhibition of TLR2 and TLR4 signaling was through interfering with the ligand-receptor interactions. Such a dual-activity of R18 relied on both cationic and hydrophobic resides in the peptide sequence. To enhance the properties of R18, the peptide was conjugated to self-assembled PEGylated phospholipid-based nanomicelles to form M-CR18. Such a nanoform of R18 displayed an improved biosafety profile and enhanced antibacterial activities with triple-destruction on bacterial biofilm formation, bacterial membrane integrity, and bacterial motility. Particularly, M-CR18 was able to down-regulate essential gene expressions responsible for the bacterial flagellar assembly, which governed the bacterial motility. In the infectious and non-infectious sepsis/ALI mouse models, M-CR18 was able to reduce the bacterial loads in the blood and major organs, reverse the lung inflammation, and increase the mouse survival rate. Moreover, M-CR18 had the ability to eradicate clinically present drug-resistant bacterial species for potential clinical applications. This study provided a *de novo* design principle for novel bioactive peptides with a dual-action on immunomodulation and bacterial killing, and presented a new generation of multifunctional nano-therapeutics for the treatment of bacterial infection-mediated pneumonia and sepsis.

## 5. Materials and Methods

*Materials:* All peptides were obtained from Nanjing Jietai Biological Company (Nanjing, China). DSPE-PEG2000-MAL was obtained from AVT (Shanghai, China). N, N-dimethylformamide (DMF) was purchased from Lian Long Bohua Pharmaceutical Chemical Company (Tianjin, China). The human monocytic THP-1 cell line was obtained from ATCC (Rockefeller, MD, USA). THP-1 reporter cell lines (XB and ISG), LPS-EK (LPS from *E. coli* K12, for cells), Poly I/C (high molecular weight, HMW), resiquimod (R848), Pam3CSK4, Zeocin, and QUANTI-Blue™ solution were purchased from InvivoGen (San Diego, CA, USA). RPMI 1640 medium, phosphate buffered saline (PBS), and fetal bovine serum (FBS) were from Biological Industries (KibbutzBeit Haemek, Israel). L-glutamine and sodium pyruvate were from Gibco (Grand Island, NY, USA). MTS assay was purchased from Promega (Madison, WI, USA). Human ELISA kits of MCP-1, TNF-α, and IL-6 were purchased from Invitrogen (Grand Island, NY, USA). Tris buffered saline (TBS), Liu stain, red blood cell (RBC) lysis buffer, and 1,1-dioctadecyl-3,3,3,3-tetramethylindodicarbocyanine (DiD) perchlorate were purchased from Solarbio Science & Technology (Beijing, China). The primary antibodies against phosphorylated p65 (#3033S) and IRF3 (#4947S), IκBα (#9242S), and β-actin (#8457S) as well as the HRP conjugated anti-rabbit (#7074S) antibodies were purchased from Cell Signaling Technology (Boston, MA, USA). The RIPA lysis buffer, Halt protease and phosphatase inhibitor cocktail were from Thermo Fisher Scientific (Waltham, MA, USA). Bovine serum albumin (BSA) was purchased from Genview (Houston, TX, USA). Tween 20, LB broth, LB broth agar and Nile Red were from Sangon Biotech (Shanghai, China). 1,1-dioctadecyl-3,3,3,3-tetramethyl lindotricarbocyaine iodide (DiR) and 2-(4-Amidinophenyl)-6-indolecarbamidine dihydrochloride (DAPI) were purchased from Beyotime (Shanghai, China). 2.5% Glutaraldehyde solution was from Solomen (Tianjin, China). Calcein-AM was purchased from USEverbright (Silicon Valley, CA, USA). DiSC3(5) was purchased from Aladdin (Shanghai, China). Mueller Hinton Broth (MHB) and Trypticase Soy Broth (TSB) were purchased from Hopebiol (Qingdao, China). DCFH-DA, dimethyl sulfoxide (DMSO) and other chemicals were obtained from Sigma-Aldrich (Sant-Louis, MO, USA) unless specified.

*Synthesis and characterization of M-CR18.* M-CR18 was fabricated with the following procedure: DSPE-PEG2000-MAL and the peptide CR18 powders with a molar ratio of 1:1.5 were dissolved in 3 mL DMF, followed by bath sonication for 5 min. The mixture was stirred in a brown bottle for 24 h, and then transferred into a dialysis bag (3500 kD) and dialyzed in half-diluted PBS for two days. After dialysis, the mixture was ultrasonicated for 10 min, filtered with a microporous membrane (0.22 µm, Millipore, Billerica, MA, USA), and stored at 4°C before use.

The size and morphology of M-CR18 was visualized using a transmission electron microscope (HT7700, Hitachi, Tokyo, Japan) with an accelerating voltage of 80 kV. The hydrodynamic diameter of M-CR18 was determined by dynamic light scattering technique on a Zetasizer instrument (Nano ZS, Malvern, Worcestershire, UK).

The critical micelle concentration (CMC) of M-CR18 was determined by pyrene fluorescence in responding to the polarity of the microenvironment 56, 57. Pyrene (6×10^-6^ M) was dissolved in acetone solution, and 100 μL of pyrene solution was transferred into a glass vial and air-dried. The diluted micelle solutions (1 mL) with conjuated peptide concentrations ranging from 0.3125 to 320 µM were added to the vial and left overnight at room temperature to allow the pyrene (6×10^-7^ M) partitioning into micelles. The fluorescence emission spectra of pyrene were collected by a fluorescence spectrophotometer with excitation at 334 nm; the intensity ratios of the first emission peak at ~373 nm (I1) over the third one at ~384 nm (I3) were plotted against the conjugated peptide concentrations to determine the CMC.

*Cell culture.* THP-1 cells were cultured in the RPMI 1640 complete medium supplemented with 10% FBS, 2 mM L-glutamine, and 1 mM sodium pyruvate, in a cell incubator containing 5% CO_2_ at 37°C. Zeocin (200 μg/mL or 100 μg/mL) was added to the complete medium as the selection pressure for THP-1-XBlue or THP-1-ISG reporter cells. Cells were seeded into a 96-well plate (1×10^5^ cells/well) or a 24-well plate (5×10^5^ cells/well), and differentiated into macrophages with PMA (50 ng/mL) for 24 h. After washing and resting for 24 h, macrophages were co-treated with R18 (or M-CR18) and different TLR agonists for 24 h: LPS (10 ng/mL) for TLR4, Pam3CSK4 (10 ng/mL) for TLR1/2, and Poly I/C (50 μg/mL) for TLR3. For TLR7/8 stimulation, THP-1 monocytes were seeded into culture plates and co-treated with R18 (or M-CR18) and R848 (10 μg/mL) for 24 h. The culture medium supernatants and cell lysates were collected for further analysis.

*Cell viability assay.* THP-1 cell viability was determined by the MTS assay. In a 96-well plate, cells were treated with M-CR18 or R18 at concentrations of 2.5, 5, 10, 20, 40 and 50 μM for 24 h, and the culture media were replaced with fresh ones (100 μL); the MTS reagent (15 μL/well) was directly added to the well and incubated at 37°C for about 2 h. The absorption at 495 nm was measured by a microplate reader (TECAN, Mannedorf, Zurich, Switzerland), and compared with that of the untreated group (100% viable).

*NF-κB/AP-1 and IRF reporter assay.* THP-1-XBlue and THP-1-ISG reporter cells were used to assess the activation of NF-κB/AP-1 and IRF, respectively. After treatments, the culture medium (20 μL per well) was collected and mixed with the Quanti-Blue solution (180 μL) in a clean 96-well plate, which was incubated for 1-2 h at 37°C until the solution color turned into dark purple. The solution absorption at 655 nm was acquired on a microplate reader (TECAN, Mannedorf, Zurich, Switzerland).

*Molecular docking of the agonists or R18 with TLR4/MD-2 and TLR1/2.* The protein structures of the TLR1/2 and TLR4/MD-2 were retrieved from the Protein Data Bank (https://www.rcsb.org/). The receptor structures were prepared based on the crystal structures of the TLR1-TLR2 heterodimer (PDB ID: 2Z7X) 58 and the human TLR4-human MD-2-*E.coli* LPS Ra complex (PDB ID: 3FXI) 59 by Molecular Operating Environment (MOE) 2019 (for the agonists) or by Protein Preparation Wizard (Release 2020-1) (for R18). For analyzing the agonists with their receptor complex, after removing water and ligands, the QuickPrep module of MOE was used to process the proteins, including protonation and energy minimization. The 2D structures of lipid A (CID: 9877306) and Pam3CSK4 (CID: 130704) were downloaded from PubChem (https://pubchem.ncbi.nlm.nih.gov/) and the WASH module of MOE was used for protonation and rebuilding 3D structures of the agonists. The Site Finder module of MOE was used to find active sites and set docking sites. The General dock module was used to manually dock protein receptors and the ligands.

For analyzing R18 with the receptor complex, the peptide structure was first constructed according to the sequence. The optimal mode of the peptide structure was adopted to determine the binding site, and then drawn by PyMOL and saved in active pdb format files. The peptide was docked into the receptor by Protein-Protein Docking (Released in 2020-1) in Standard mode with default parameters. The binding ΔG of the peptide with the receptors were calculated by MM-GBSA (Release 2020-1) and the best scored pose was chosen. The binding-site surface of the receptors was truncated at 3Å from the peptide, generated by Maestro (Released in 2020-1). The docking results were visually analyzed using UCSF Chimera 60.

*Surface plasmon resonance analysis.* The binding of R18 with the TLR4/MD-2 complex and TLR2 (R&D Systems, Minneapolis, MN, USA) were examined using Biacore 8K (Cytiva, Sweden). The TLRs were covalently immobilized onto two different flow cells of a CM5 chip using a standard EDS/NHS amine coupling method with 10 mM sodium acetate buffer (pH 4.5) for TLR4/MD-2 or an equal ratio of pH 4.5 and pH 5.0 sodium acetate solution (10 mM) for TLR2. One flow cell served as a reference and was immediately blocked with 1 M ethanolamine. In the other flow cells, the TLR4/MD-2 complex or TLR2 (25 μg/mL) was injected to a CM5 chip surface with a resonance value of about 8000. R18 at different concentrations (0.039, 0.078, 0.156, 0.313 and 0.625 μM) in PBS containing 5% DMSO and 0.05% Tween-20 was injected into the reference flow cell and the TLR4/MD-2 complex or TLR2-immobilized flow cell. The binding curves of R18 with TLR4/MD-2 complex and TLR2 were fitted by Biacore 8K Evaluation Software 3.0 after correction with the DMSO standard curve.

*Measurement of cytokines.* The pro-inflammatory cytokines IL-6 and TNF-α in the culture medium or in the BALF were quantified by ELISA according to the manufacturing instructions.

*Immunoblotting.* The THP-1 cell-derived macrophages were stimulated with LPS (10 ng/mL) with or without R18 and M-CR18 treatment for different time period (0.5, 1, and 2 h). After washing with PBS, cells were lysed by ice-cold RIPA buffer supplemented with Halt protease and phosphatase inhibitor cocktail. The total protein concentration was quantified by the Bradford assay kit (Coomassie Plus, Thermo Fisher Scientific, Waltham, MA, USA) and adjusted accordingly. The proteins were separated by 10% SDS-PAGE and then transferred to a PVDF membrane (Immobilon-P, Millipore, Billerica, MA, USA). After blocking with 5% BSA in the TBS buffer containing 0.1% Tween 20 for at least 1 h at room temperature, the membranes were blotted with primary antibodies against phosphorylated p65 and IRF3, IκBα, and β-actin overnight at 4°C; they were then incubated with the HRP-conjugated anti-rabbit secondary antibody for 1 h at room temperature. The protein bands were imaged using the chemiluminescence method (ECL, Millipore, Billerica, MA, USA) on a ChemiDoc MP imaging system (Bio-Rad, Hercules, CA, USA).

*Bacterial culture.* Three representative strains of Gram-negative bacteria were used in this study: *E. coli* DH5-α was obtained from Tiangen (Beijing, China); *P. aeruginosa* (PA103) was provided by Professor Ning Wen's research group at Nankai University; the standard strain of *E. coli* K12 (MG1655) was obtained from Professor Wang Quan at Tianjin Medical University. The bacteria were grown in the LB medium at 37°C under shaking for about 6 h for experiments, and the LB agar plates were used for bacterial colony counts. The clinically isolated drug-resistant bacterial strains were obtained from the group of Professor Yuanlin Song at Zhongshan Hospital, Fudan University.

*Antibacterial assessment in vitro.* The broth microdilution method was used to determine the minimum inhibitory concentration (MIC). A single bacterial colony was cultured in the LB broth medium at 37°C, under continuous shaking (160 rpm) for 6 h until the growth of bacteria reached log phase (with an OD value between 0.6-0.8 at 600 nm). The concentration of the bacterial suspension was adjusted to 1.5×10^8^ CFU/mL according to the growth curve and then diluted to 1×10^5^ CFU/mL. Mueller-Hinton broth (MHB, 100 μL) was added to each well of a 96-well plate, and the antibacterial agents (100 μL) (M-CR18 or R18) were added to the first well, followed by two-fold dilution to the consecutive wells to have the peptide concentrations in each well: 40, 20, 10, 5, 2.5, 1.25, 0.625, and 0.3125 μM. The bacterial suspensions (100 μL) were then added into each well, and incubated at 37°C for 20 h. The blank broth without bacteria and the untreated bacterial suspensions were used as negative and positive controls, respectively. The turbidity was assessed by measuring OD values at 600 nm on a microplate reader (TECAN, Mannedorf, Zurich, Switzerland), and the MIC values were determined accordingly. Aliquots of the mixtures were taken from each well for inoculation on the surface of the LB agar plate and cultured at 37°C for 16 h to confirm the MIC experiment and to determine the minimum bactericidal concentration (MBC).

*Examination of Biofilm disruption.* The biofilm inhibition was assessed by the crystal violet staining experiment. The *E. coli* culture (1×10^5^ CFU/mL, 100 μL) was prepared in MHB medium in a 96-well plate mixed with a serial concentration of the antibacterial agents following the above dilution method. After incubation at 37°C for 1 day, the culture medium was discarded, and the plates were washed three times with PBS to remove planktic bacteria. Biofilms from each well were fixed with 95% ethanol for 15 min, and treated with 0.1% crystal violet (100 μL). The plates were rested in the dark for 20 min, and the stained biofilms were washed three times with PBS and air-dried. Acetic acid (33% v/v, 100 μL) was added to each well to dissolve the stained crystals, and the OD at 560 nm was acquired on a microplate reader (TECAN, Mannedorf, Zurich, Switzerland) to evaluate the biofilm inhibition.

The biofilm formation was also characterized by the scanning electronic microscopy (SEM). *E. coli* was cultured overnight and diluted to 1×10^5^ CFU/mL in the LB broth. The antibacterial agents (300 μL) at different concentrations (5, 20, and 80 μM) were mixed with equal volume of bacterial suspensions in a 24-well plate with a clean silicon wafer at the bottom of the well. The plate was incubated at 37°C for 48 h; at 24 h, the culture medium was replaced with a fresh one. The bacteria were rinsed with PBS three times and fixed with 2.5% glutaraldehyde for 18 h at 4 °C. The samples were washed with PBS, dehydrated in an increased gradient of ethanol solution (30-100%), and air-dried at room temperature; they were then processed with gold coatings prior to the SEM imaging (Gemini 300, Zeiss, Oberkochen, Germany).

Confocal fluorescence imaging was applied to characterize the biofilm formation as another complementary method. Equal volumes of *E. coli* suspensions (1×10^5^ CFU/mL) and antibacterial agents (10 μM) of M-CR18 and R18 were mixed and incubated at 37°C for 24 h in a confocal cell dish (NEST, Wuxi, China). The culture medium was then replaced without the presence of M-CR18 and R18. After another 24 h incubation, the culture dish was washed three times with PBS to remove the planktons. The biofilms were stained with Calcein-AM (500 μL, 30 μM) for 30 min and washed twice with PBS. The samples were imaged on a confocal laser scanning microscope (LSM900, Leica, Wetzlar, Heessen, Germany) with excitation at 490 nm and emission at 515 nm.

*Investigation on the bacterial membrane potential.* The changes in the bacterial membrane potential were examined by the zeta potential measurement and the potentiometric probe DiSC3(5). The diluted *E. coli* suspensions (1×10^5^ CFU/mL) were mixed with equal volume of different peptide concentrations (80, 20, and 5 μM) of M-CR18 and R18. After incubation at 37℃ for 2.5 h, the zeta potential was measured on a Zetasizer (Nano ZS, Malvern, Worcestershire, UK). For DiSC3(5) staining, the bacterial suspensions were added into a 96-well black plate and stained with DiSC3(5) (4 μM) for 30 min. The samples were mixed with different concentrations of the antibacterial agents and incubated 37℃ for 2.5 h. The fluorescence (ex: 620 nm; em: 670 nm) of the samples was detected on a microplate reader (TECAN, Mannedorf, Zurich, Switzerland). The average biofilm thickness was quantified by Biofilm Analysis plugin in the IMARIS (Bitplane, Zurich, Switzerland) 61, 62.

*The affinity of R18 and M-CR18 with the bacterial membrane. E. coli* suspensions (3×10^8^ CFU/mL) were treated with DiO-labeled M-CR18 (10 μM) or FITC-labeled R18 (20 μM) for 4 h. After washing with PBS, the mixtures were stained with Nile Red (5 μM) in the dark at 37℃ for 15 min. They were then washed with PBS and fixed with 4% paraformaldehyde for 10 min. The fixed bacterial suspensions (5 μL) were transferred to a sterilized glass slide. The fluorescence of Nile red (ex: 552 nm; em: 636 nm), FITC (ex: 485 nm; em: 530 nm) and DiO (ex: 484 nm; em: 501 nm) were imaged on a confocal laser scanning microscope (LSM800, Leica, Wetzlar, Hessen, Germany).

*In vivo studies.* C57BL/6 wild-type male mice (6-8 weeks) were obtained from SPF Biotechnology Co., Ltd. (Beijing, China). All mice were kept in the Tianjin Medical University animal laboratory center (SPF level) or raised in a SPF facility in Naikai University prior to experiments. The animal experiment procedures for mouse handling, and the LPS-induced ALI and CLP-induced sepsis mouse models were approved by the Animal Care Committee of Tianjin Medical University (Approval No: TMUaMEC2020004). The animal experiment protocols for *P. aeruginosa*-induced ALI mouse model were approved by the Animal Care Committee of Nankai University (Approval No. 20140008).

*LPS-induced ALI mouse model.* The ALI mouse model was established by intratracheal administration of LPS (10 mg/kg) for 24 h. M-CR18 (30 nmol/kg) was given intratracheally 2 h before LPS challenge. Mice were sacrificed to collect the BALF and lung tissues for subsequent analysis on cell infiltration, cytokine production and lung injury severity. All procedures were performed under 1% sodium pentobarbital (45 mg/kg) anesthesia via intraperitoneal injection.

*The cecal ligation and puncture (CLP)-induced sepsis mouse model.* The CLP mouse model was employed to mimic the clinical condition of bacterial infection-induced sepsis and ALI. Under 1% pentobarbital sodium (45 mg/kg) anesthesia, mice were under surgery with a longitudinal incision (~2 mm) in the middle of the abdomen to locate the cecum. It was ligated by sterile sutures at half of the cecum distal to the ileocecal valve, and the ligated cecum was perforated with a 22G needle. The cecum was gently squeezed to let the feces out of the hole, and the cecum was then put back into the abdominal cavity. After the peritoneum and skin were sutured, pre-warmed (37°C) 0.9% saline (1 mL) was injected subcutaneously into the back of mice. Mice were treated with M-CR18 (120 nmol/kg) or the same volume of PBS as control intrapertoneally at 20 min and 72 h after the surgery. Mouse survival rate was recorded for up to 15 days. The protective effects of M-CR18 were assessed in mice with mild sepsis by CLP induction, where the ligation was done at 1/3 of the mouse cecum after midline laparotomy. The PBS or M-CR18 (120 nmol/kg) via i.p. injection 20 min after CLP, and the blood, liver, spleen, heart, lung, and kidney were harvested 3 days after for the quantification of bacterial loads and for assessing lung injury.

*Pseudomonas aeruginosa-induced ALI mouse model.* In addition to CLP model, mice were anesthetized with avertin (Sigma-Aldrich, Sant-Louis, MO, USA) and then intratracheally injected with *P. aeruginosa* (strain PA103) at a dose of 1.5×10^6^ CFU per mouse for the analysis of the survival rate and body temperature; a lower dose of 1.0×10^6^ CFU per mouse was applied directly to the lung to establish the *P. aeruginosa* lung infection mouse model for the analysis of the inflammatory responses in the lung. M-CR18 (360 nmol/kg) was administered via the same route when challenged with PA103.

*BALF collection and differential cell count.* At the end of ALI model, the mice underwent tracheotomy, and sterile PBS (0.8 mL) was injected intratracheally into the lungs twice. The BALF was collected 30 s after each injection and centrifuged at 1000 rpm for 10 min at 4 °C. The supernatant was collected and stored at -80 °C for cytokine analysis. BAL cells were treated with 4% red cell lysis buffer and resuspended in PBS. The total cell counts were performed on a hemocytometer. Aliquots of the cell suspensions were spun down on a glass slide by a Cytospin (Thermo Fisher Scientific, Waltham, MA, USA), which was processed with Liu's staining and observed on a microscope (ECLIPSE NI-U, Nikon, Tokyo, Japan) for differential cell counting. At least 300 cells were counted for each sample.

*Lung histology and injury scoring.* The left lung of mice was collected, fixed with 4% paraformaldehyde, dehydrated, embedded in the paraffin, and cut into 5-μm thick sections. The tissue sections were stained with hematoxylin and eosin (H&E) and imaged on a microscope (ECLIPSE NI-U, Nikon, Tokyo, Japan). For each sample, at least 20 fields (400×) were scored blindly by three independent investigators on five histopathological features including alveolar neutrophils, interstitial neutrophils, hyaline membranes, protein fragments, and alveolar septal thickening 63.

*Biodistribution of M-CR18.* Mice were intratracheally administered with DiR-labeled M-CR18 (125 nmol/kg) 1 h prior to the LPS (5 mg/kg) challenge via the same route. Mice were sacrificed 1 day or 1 week post LPS challenge. Major organs/tissues including the lungs, heart, liver, spleen, kidneys, and gastrointestinal track as well as the blood were collected. These samples were imaged using an IVIS system (IVIS SPECTRUM, PE, MA, USA) with excitation at 740 nm and emission at 800 nm.

*Bacterial RNA sequencing. E. coli* (K12, 2×10^7^ CFU/mL) suspensions were treated with M-CR18 or R18 (80 μM) at 37℃ for 4 h, and the total RNA was extracted using Total RNA Extraction Kit (Tiangen, Beijing, China). After confirming the purity and concentration of the extracted RNA by Nanodrop (ALLSHENG, Hangzhou, China), the samples were sent to Novogene (Beijing, China) for high-throughput RNA sequencing. The differentially expressed genes were selected by setting padj < 0.05 and log|FC| > 1 as the thresholds, and the gene expression levels were clustered using the R package pheatmap for heat map analysis. The results of p-value < 0.05 in the up- and down-regulated genes were displayed using the R package ggplot2 for the volcano plot as well as the KEGG pathway analysis. Gene expression on specific pathways was demonstrated using the R package GOplot.

The expressions of the genes of interest were confirmed by qRT-PCR. The extracted bacterial total RNA was first reverse transcribed to cDNA. The expressions were then quantified on a real-time PCR system using the SYBR Green kit. The expression profile was normalized with the internal reference gene *rrsA*. All primer sequences used were listed in **Table S3**.

*Bacterial flagellin formation by TEM. E. coli* (K12, 2×10^7^ CFU/mL) suspensions were mixed with M-CR18 or R18 (80 μM) at 37℃ for 4 h. They were centrifuged (8000 rpm, 5 min) and fixed using 4% paraformaldehyde for 20 min. Aliquots (20 μL) of resuspended bacterial suspensions in PBS were deposited on a copper grid and rested for 30 min, and the samples were stained with 0.5% phosphotungstic acid for 10 min. After air-dry, the samples were imaged on a TEM (HT7700, Hitachi, Tokyo, Japan) to visualize the bacterial flagellin.

*Bacterial motility assay.* The 0.5% agar plate was prepared to assess the bacterial motility. The agar solution was made of 1 g peptone, 0.5 g yeast extract, 0.5 g agar powder and 0.5 g sodium chloride in 100 mL ultrapure water, which was sterilized and slowly cooled down at room temperature. Prior to solidification, the agar liquid was mixed with R18 or M-CR18 with a final concentration of 80 μM (200 μM for PA103), and transferred into a 6-well plate (5 mL/well) until fully solidified at room temperature. Aliquots (1 μL) of *E. coli* (K12, 2 × 10^7^ CFU/mL) or *P. aeruginosa* (PA103, 2 × 10^4^ CFU/mL) suspensions were added to the center of the agar and cultured at 37°C for 15 h. The spread of the bacterial spot from the center was observed, and the largest diffusion diameter across the colony was quantified to determine the bacterial motility.

*Analysis of TLR expression in septic patients.* The analysis was based on the gene microarray data from the GEO database (https://www.ncbi.nlm.nih.gov/pmc/). The data of adult sepsis samples were downloaded from the dataset (GSE69063) with the gene microarray data type of Expression profiling by array, the species of Homo sapiens, and the data annotation platform of GPL19983, which included 19 patients with newly admitted sepsis, 20 patients with sepsis at 1 h of admission, 18 patients with sepsis at 3 h of admission, and 33 normal controls in this dataset. The data from the pediatric sepsis samples were downloaded from the dataset (GSE26378) with the gene microarray data type of Expression profiling by array, the species of Homo sapiens, and the data annotation platform of GPL570. The dataset consisted of 82 pediatric sepsis patients and 21 normal controls.

The GEO data were read, normalized and analyzed using R software 4.2.1. The Limma package was used to statistically analyze the differential genes between septic patients and the normal controls in both datasets; the differential genes were screened with the threshold of difference multiplicity |log2FC| > 1 and p-value < 0.05 for the expressions of *TLR2* and *TLR4* and related inflammatory genes. The R language software ggplot and pheatmap packages were used to plot heat maps and dot plots of differentially expressed genes. Pearson correlation analysis was applied to assess the correlation between the expression of the inflammatory gene *IL1B* and *TLR2/4* genes.

*Determination of MIC/MBC of R18/M-CR18 on clinical drug-resistant bacterial strains.* The ethical approval was obtained from the Ethics Committee at Zhongshan Hospital Affiliated to Fudan University (B2022-044R). The antimicrobial effects of R18/M-CR18 on *P. aeruginosa* MDR and XDR clinical strains were performed by microdilution broth sensitivity test as previously described with minor modifications 64, 65. Briefly, the antibacterial agents with different diluted concentrations (4.7, 9.4, 18.8, 37.5, 75, 150 and 300 μM) were mixed with 1×10^5^ CFU/mL of drug-resistant bacteria in LB broth and transferred into a 96-well plate under incubation at 37°C for 24 h; the MIC value was determined by the wells with the lowest concentration, where no visible bacterial growth was observed. For MBC determination, bacteria were mixed with the agents at different concentrations and poured into blood agar plates. After incubating for 24 h at 37°C, the MBC value was defined as the minimum concentration without observed bacterial colony on the plate.

*Statistical analysis.* All statistical analyses were performed using GraphPad Prism 7.0. All data were presented as mean ± standard error of mean (SEM). P-value < 0.05 was considered statistically significant. Student t-test was used for the comparison between two groups whereas one-way ANOVA with Bonferroni post-test was applied for multiple comparisons between groups.

## Supplementary Material

Supplementary: Additional figures describe the up-regulated expressions of *TLR2* and *TLR4* in septic patients, the effects of R18 on TLR4- and TLR2-mediated cytokine production, TLR3 and TLR7/8 signaling pathways in THP-1 cell-derived macrophages, the binding of R18/M-CR18 to TLR2/4 ligands, TLR2/4 inhibition by R18/M-CR18 with the pre-treatment and wash procedure, MIC and MBC of R18 and its derivatives WA4 and LA6 on *E. coli*, the quantitative analysis of M-CR18 size from TEM images, pyrene fluorescence spectra at different M-CR18 concentrations, the cytotoxicity of R18 and M-CR18 in the endothelial cell line Eahy-926, the effects of R18 and M-CR18 on different inflammatory signaling pathways and TLR4- and TLR2-mediated IL-1β production in THP-1 reporter cell-derived macrophages, the effects of R18 and M-CR18 on the formation of bacterial flagella of *E. coli*, the effects of R18 and M-CR18 on the bacterial motility of *P. aeruginosa*, the scoring of five histopathological features of the injured lungs in the LPS-induced ALI mouse model, the effects of M-CR18 on the bacterial load in the vital organs and on the pathological scores of injured lungs in the CLP-induced mild sepsis mouse model, the biosafety profile of M-CR18, and the effects of R18 and M-CR18 on various clinical antibiotics-resistant bacterial strains.
