# Supplementary material for: Designer amphiphilic helical peptide-decorated nanomicelles enable simultaneous inflammation control and triple-destruction of bacteria for treating bacterial pneumonia and sepsis
Source: Theranostics. 2025 Aug 16;15(17):9047–72. doi: 10.7150/thno.110538 (PMC12439338; doi:10.7150/thno.110538)
Supplement: Supplementary file 1 — Supplementary: Additional figures describe the up-regulated expressions of TLR2 and TLR4 in septic patients, the effects of R18 on TLR4- and TLR2-mediated cytokine production, TLR3 and TLR7/8 signaling pathways in THP-1 cell-derived macrophages, the binding of R18/M-CR18 to TLR2/4 ligands, TLR2/4 inhibition by R18/M-CR18 with the pre-treatment and wash procedure, MIC and MBC of R18 and its derivatives WA4 and LA6 on E. coli, the quantitative analysis of M-CR18 size from TEM images, pyrene fluorescence spectra at different M-CR18 concentrations, the cytotoxicity of R18 and M-CR18 in the endothelial cell line Eahy-926, the effects of R18 and M-CR18 on different inflammatory signaling pathways and TLR4- and TLR2-mediated IL-1β production in THP-1 reporter cell-derived macrophages, the effects of R18 and M-CR18 on the formation of bacterial flagella of E. coli, the effects of R18 and M-CR18 on the bacterial motility of P. aeruginosa, the scoring of five histopathological features of the injured lungs in the LPS-induced ALI mouse model, the effects of M-CR18 on the bacterial load in the vital organs and on the pathological scores of injured lungs in the CLP-induced mild sepsis mouse model, the biosafety profile of M-CR18, and the effects of R18 and M-CR18 on various clinical antibiotics-resistant bacterial strains. [file thnov15p9047s1.pdf]

Supporting Information for

Designer amphiphilic helical peptide-decorated nanomicelles enable simultaneous inflammation control and triple-destruction of bacteria for treating bacterial pneumonia and sepsis

Sixia Liu[1,#], *Rui Wang*[1,#], *Lian Li*[2,#], *Xiaohuan Wang*[3], *Jiameng Gong*[1], *Xingzu Liu*[2], *Zichen Song*[4], *Liya Sun*[1], *Xiali Liu*[5], *Wen Ning*[2], *Yuanlin Song*[6,*], *Shan-Yu Fung*[7,*] and *Hong Yang*[1,*]

[1]Tianjin Key Laboratory of Inflammation Biology, The Province and Ministry Co-Sponsored Collaborative Innovation Center for Medical Epigenetics, Department of Pharmacology, School of Basic Medical Sciences, Intensive Care Unit of the Second Hospital, Tianjin Medical University, Tianjin 300070, China

[2]State Key Laboratory of Medicinal Chemical Biology, College of Life Sciences, Tianjin Key Laboratory of Protein Sciences, Nankai University, Tianjin 300071, China.

[3]Department of Laboratory Medicine, Zhongshan Hospital, Fudan University, Shanghai 200032, China.

[4]Department of Anesthesia, Tianjin Institute of Anesthesiology, Tianjin Medical University General Hospital, Tianjin 300052, China

[5]Department of Pulmonary and Critical Care Medicine, Shanghai General Hospital, Shanghai Jiao Tong University School of Medicine, Shanghai 201620, China

[6]Department of Pulmonary and Critical Care Medicine, Zhongshan Hospital, Fudan University, Shanghai 200032, China.

[7]State Key Laboratory of Experimental Hematology, Key Laboratory of Immune Microenvironment and Disease (Ministry of Education), Department of Immunology, School of Basic Medical Sciences, Tianjin Medical University, Tianjin 300070, China

#These authors contributed equally to the paper.

*Corresponding authors:

Professor Hong Yang (E-mail: hongyang@tmu.edu.cn)

Professor Shan-Yu Fung (Email: shanefung@tmu.edu.cn)

Professor Yuanlin Song (E-mail: ylsong70@163.com; song.yuanlin@zs-hospital.sh.cn)

The PDF file includes the following materials:

1. Supplementary materials and methods

1.1. Materials

1.2. Measurement of cytokines

1.3. Fluorescence polarization

1.4. Cell viability assay

1.5. Prediction and analysis of the secondary structure of R18

2. Supplementary figures

Figure S1 The up-regulated expressions of TLR2 and TLR4 genes in sepsis patients.

Figure S2 The effects of R18 on TLR4- and TLR2-mediated MCP-1 and TNF-α production in THP-1 cell-derived macrophages.

Figure S3 The effects of R18 on TLR3 and TLR7/8 signaling pathways.

Figure S4 The binding of R18 to TLR2/4 ligands.

Figure S5 The inhibition of TLR2/4 activation by R18/M-CR18 with the pre-treatment and wash procedure.

Figure S6 MIC and MBC of R18 and its derivatives WA4 and LA6 on *E. coli*.

Figure S7 The quantitative analysis of the diameter of M-CR18 from TEM images.

Figure S8 Pyrene fluorescence spectra at different concentrations of M-CR18.

Figure S9 Cytotoxicity of R18 and M-CR18 in the endothelial cell line Eahy-926.

Figure S10 The effects of R18 and M-CR18 on different inflammatory signaling pathways in THP-1 reporter cell-derived macrophages.

Figure S11 The effects of R18 and M-CR18 on the TLR4- and TLR2-mediated IL-1β production in THP-1 cell-derived macrophages.

Figure S12 The binding of M-CR18 to TLR4 ligand.

Figure S13 The enriched Gene Ontology (GO) analysis of the transcriptome of M-CR18 treated *E. coli*.

Figure S14 The effects of R18 and M-CR18 on the formation of bacterial flagella of *E. coli*.

Figure S15 The effects of R18 and M-CR18 on the bacterial motility of *P. aeruginosa*.

Figure S16 The scoring of five histopathological features of the injured lungs in the LPS-induced ALI mouse model.

Figure S17 The effects of M-CR18 on the bacterial load in the vital organs and on the pathological scores of injured lungs in the CLP-induced mild sepsis mouse model.

Figure S18 The biosafety profile of M-CR18 in healthy mice.

Figure S19 The effects of R18 and M-CR18 on various clinical antibiotics-resistant bacterial strains.

3. Supplementary tables

Table S1 Prediction and analysis of the α-helix structure of R18.

Table S2 The binding free energy ($\Delta G_{binding}$) of TLR ligands and R18 with the receptors.

Table S3 The primer sequences used for qRT-PCR.

1. Supplementary methods

1.1. Materials

Human ELISA kits (MCP-1, IL-1β) were purchased from Invitrogen (Grand Island, NY, USA). Endothelial cells Eahy-926 was kindly provided by Professor Wen Ning's research group at Nankai University.

1.2. Measurement of cytokines

The concentrations of the pro-inflammatory cytokines MCP-1 and IL-1β in the culture medium or in the BALF were measured by ELISA according to the manufacturing instructions.

1.3. Fluorescence polarization

Fluorescence polarization was applied to examine the binding of R18 with LPS and Pam3CSK4. R18 was dissolved in the tris buffer (pH = 9.0) at various concentrations: 0.625, 1.25, 2.5, 5, 10, 20, 40, 80, 160, 320, 400 and 500 μM. The FITC-labeled LPS (10 ng/mL) was mixed and incubated with different concentrations of R18 or M-CR18 for 30 min at room temperature in a 96-well plate. For Pam3CSK4 binding assay, R18/M-CR18 at different concentrations (3.9, 7.8, 15.6, 31.25, 62.5,125, 250, 500, 1000 and 2000 μM) were mixed with the FITC-labeled Pam3CSK4 (10 ng/mL) and incubated at the same conditions as LPS. For LTA binding assay, LTA at different concentrations (3.9, 7.8, 15.6, 31.25, 62.5, 125, 250, 500, 1000, 2000 and 4000 μM) were mixed with FITC-labeled R18 (20 μM) and incubated at the same conditions as LPS. The fluorescence polarization of FITC was measured (ex: 485 nm; em: 530 nm) on a microplate reader (TECAN, Mannedorf, Zurich, Switzerland). The data was fitted using GraphPad Prism7 software to obtain the dissociation constant (Kd).

1.4. Cell viability assay

Endothelial cells Eahy-926 viability was determined by the MTS assay. In a 96-well plate, cells were treated with M-CR18 or R18 at concentrations ranging from 2.5 to 50 μM (2.5, 5, 10, 20, 40, 50) for 24 h, and the culture media were replaced with fresh ones (100 μL); the MTS reagent (15 μL/well) was directly added to the well and incubated at 37°C for about 2 h. The absorption at 495 nm was measured by a microplate reader (TECAN, Mannedorf, Zurich, Switzerland), and compared with that of the untreated group (100% viability).

1.5. Prediction and analysis of the secondary structure of R18

Peptide secondary structure prediction and analysis were performed based on the peptide amino acid sequence using different online web sources: PredictProtein (https://predictprotein.org/), BesSel (https://bestsel.elte.hu/index.php) and K2D3 (http://cbdm-01.zdv.uni-mainz.de/~andrade/k2d3/).

2. Supplementary Figures

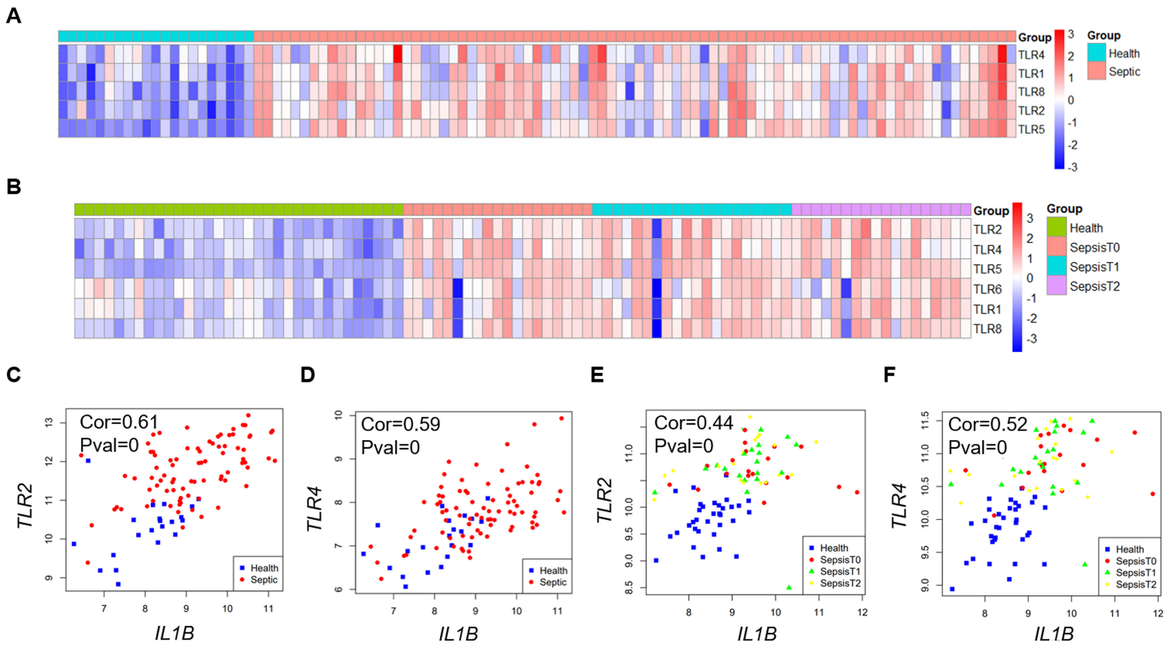

Figure S1. The up-regulated expressions of TLR2 and TLR4 genes in sepsis patients. (A, B) Heat map of TLR gene expressions in samples from pediatric sepsis patients (A) and adult sepsis patients at different disease stages (T0: newly admission, T1: 1 h admission, T2: 3 h admission) (B). (C-F) Scatter plots showing the correlation of the gene expression of *TLR2* (C, E) and *TLR4* (D, F) with that of *IL1B* in pediatric sepsis patients (C, D) and adult sepsis patients (E, F); blue dots represented healthy controls.

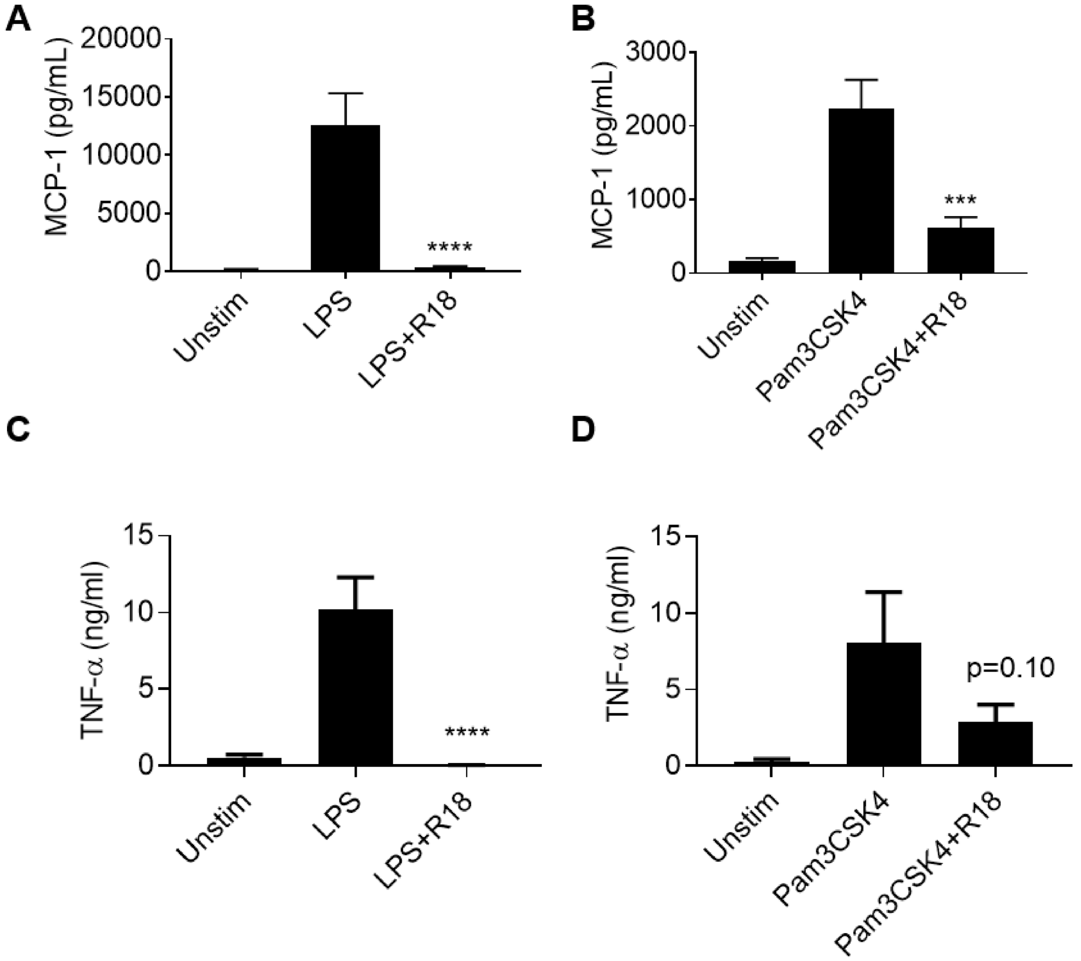

Figure S2. The effects of R18 on TLR4- and TLR2-mediated MCP-1 and TNF-α production in THP-1 cell-derived macrophages. (A, B) The production of cytokine MCP-1 was inhibited by R18 under LPS (A) and Pam3CSK4 (B) stimulation. (C, D) The production of cytokine TNF-α was inhibited by R18 under LPS (C) and Pam3CSK4 (D) stimulation. N = 3; R18 = 10 μM, LPS = 10 ng/mL, Pam3CSK4 = 10 ng/mL. The data is presented as the mean ± SEM. ***$p < 0.001$, ****$p < 0.0001$ vs. the stimulation group.

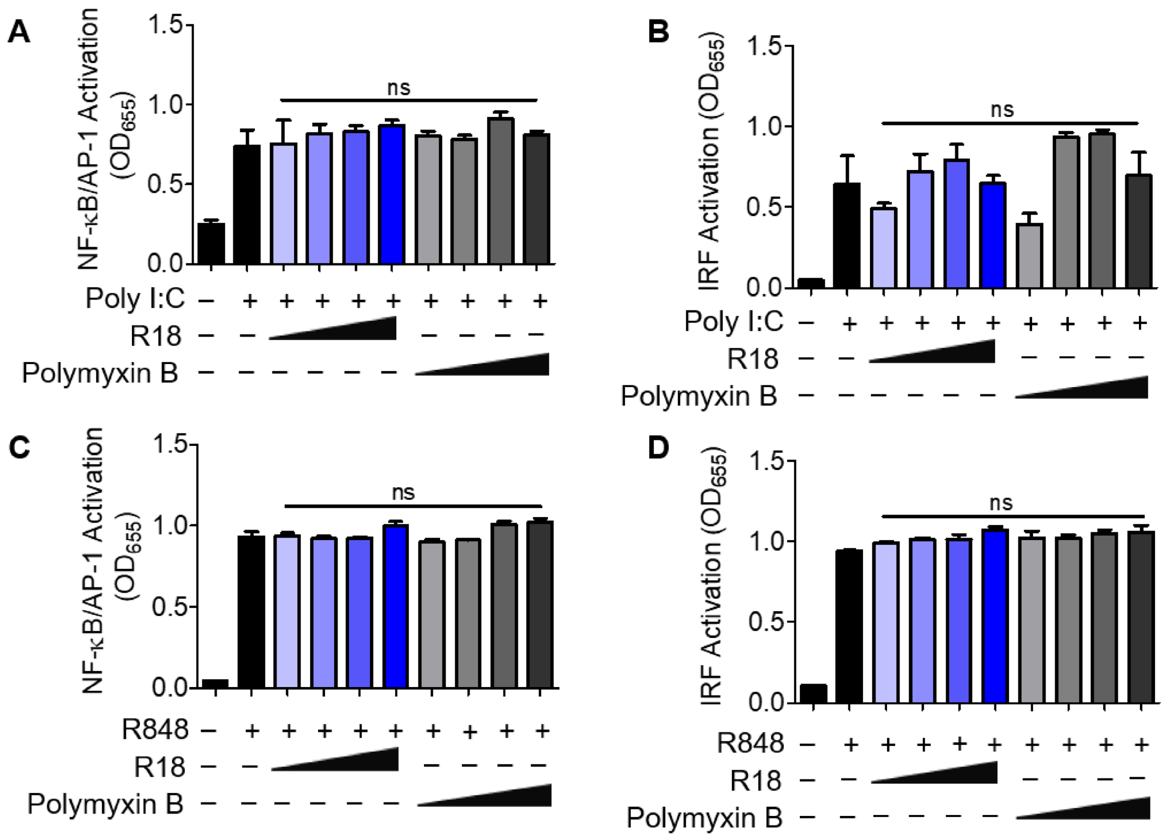

Figure S3. The effects of R18 on TLR3 and TLR7/8 signaling pathways. (A, B) Effects of R18 and polymyxin B on NF-κB/AP-1 (A) and IRF (B) activation under PolyI/C (50 μg/mL) stimulation (for TLR3) in THP-1 reporter cell-derived macrophages. (C, D) Effects of R18 and polymyxin B on NF-κB/AP-1 (C) and IRF (D) activation upon R848 (10 μg/mL) stimulation (for TLR7/8) in THP-1 reporter cell-derived macrophages. N = 3; R18 and polymyxin B = 1.25, 2.5, 5, 10 μM. The data is presented as the mean ± SEM. ns: not significant vs. the stimulation group.

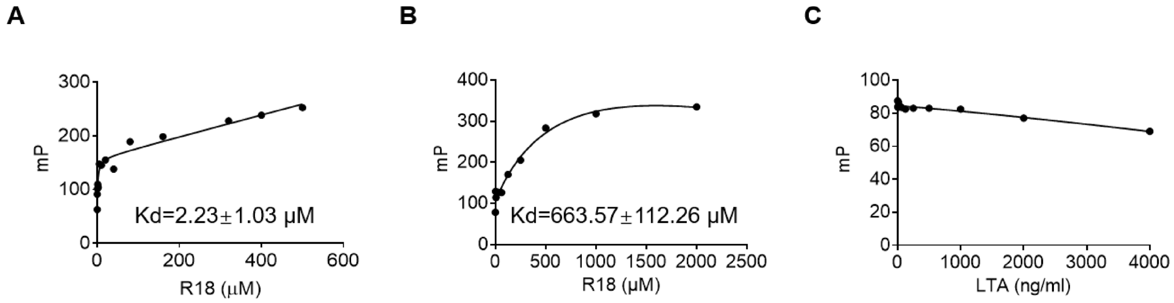

Figure S4. The binding of R18 to TLR2/4 ligands. (A, B) The fluorescence polarization profile of FITC-labeled LPS (10 ng/mL) (A) and FITC-labeled Pam3CK4 (10 ng/mL) (B) mixed with various concentrations of R18; the dissociation constant Kd was obtained from the non-linear fitting of the curve. (C) The fluorescence polarization profiles of FITC-labeled R18 (20 μM) mixed with various concentrations of LTA.

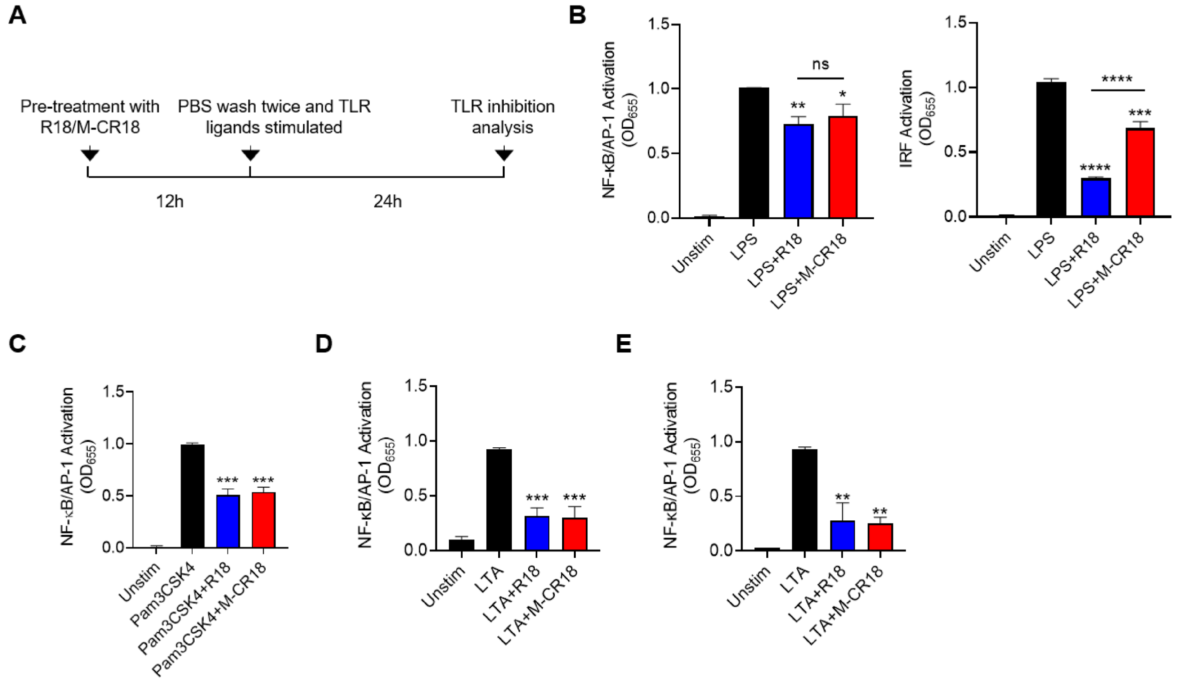

Figure S5. The inhibition of TLR2/4 activation by R18/M-CR18 with the pretreatment and wash procedure. (A) The schematic diagram of the R18/M-CR18 pre-treatment and wash experimental procedure. (B) The inhibition of LPS-induced NF-κB/AP-1 (left) and IRF (right) activation by the R18/M-CR18 pre-treatment in THP-1 reporter cell-derived macrophages; LPS = 10 ng/mL, R18 and M-CR18 = 5 μM. (C) The inhibition of Pam3CSK4-induced NF-κB/AP-1 activation by the pre-treatment of R18/M-CR18 in THP-1 reporter cell-derived macrophages; Pam3CK4 = 10 ng/mL, R18 and M-CR18 = 5 μM. (D, E) The inhibition of LTA-induced NF-κB/AP-1 activation after co-treatment of R18/M-CR18 with LTA (D) or pre-treatment of R18/M-CR18 (E) in THP-1 reporter cell-derived macrophages; LTA = 1 μg/mL, R18 and M-CR18 = 5 μM. The data is presented as the mean ± SEM. ns: not significant, *p < 0.05, **p < 0.01, ***p < 0.001, ****p < 0.0001 vs. the stimulation group unless otherwise indicated.

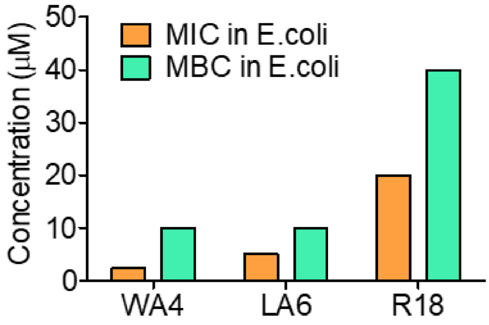

Figure S6. MIC and MBC of R18 and its derivatives WA4 and LA6 on *E. coli*. These experiments were repeated three times independently, and the same MIC and MBC were obtained.

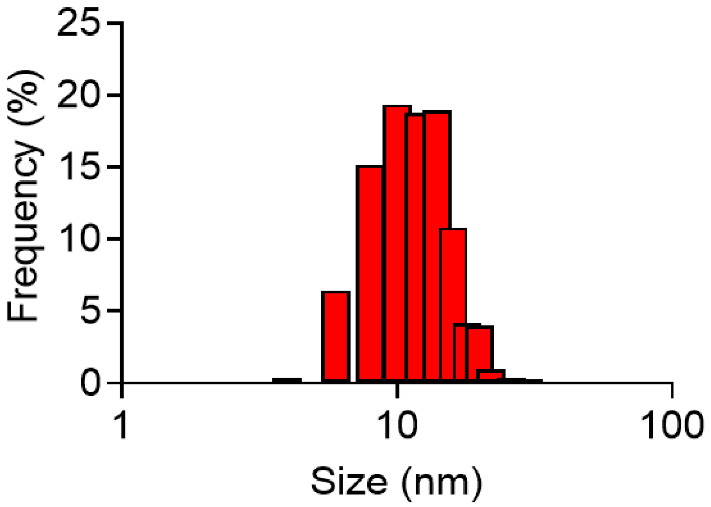

Figure S7. The quantitative analysis of the diameter of M-CR18 from TEM images.

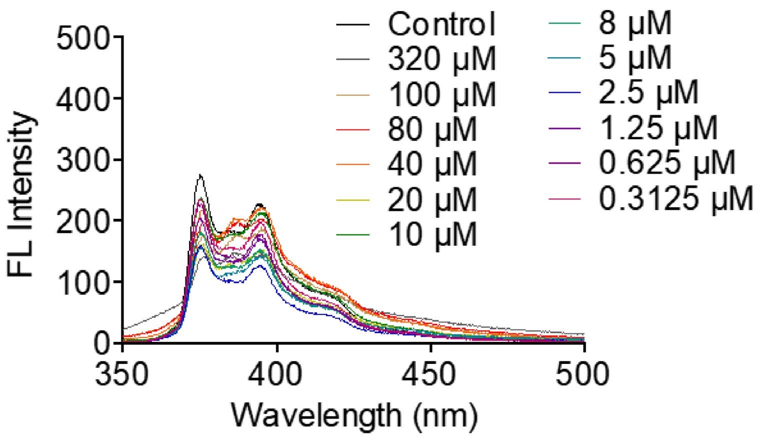

Figure S8. Pyrene fluorescence spectra at different concentrations of M-CR18. Pyrene emission fluorescence was acquired from 350 nm to 500 nm. The intensity ratios of the first peak (I1) versus the third peak (I3) at each M-CR18 concentration was obtained from the spectrum to determine the critical micelle concentration (CMC) of M-CR18.

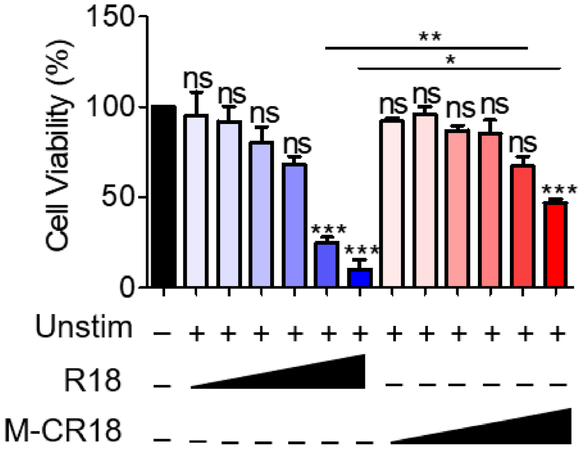

Figure S9. Cytotoxicity of R18 and M-CR18 in the endothelial cell line Eahy-926. The endothelial cells were treated with R18 or M-CR18 at different concentrations for 24 h. The cell viability was examined by the MTS assay. M-CR18 and R18 = 2.5, 5, 10, 20, 40, 50 μM; N = 3. The data is presented as the mean ± SEM. ns: not significant, *p < 0.05, **p < 0.01, ***p < 0.001 vs. the unstimulated control unless otherwise indicated.

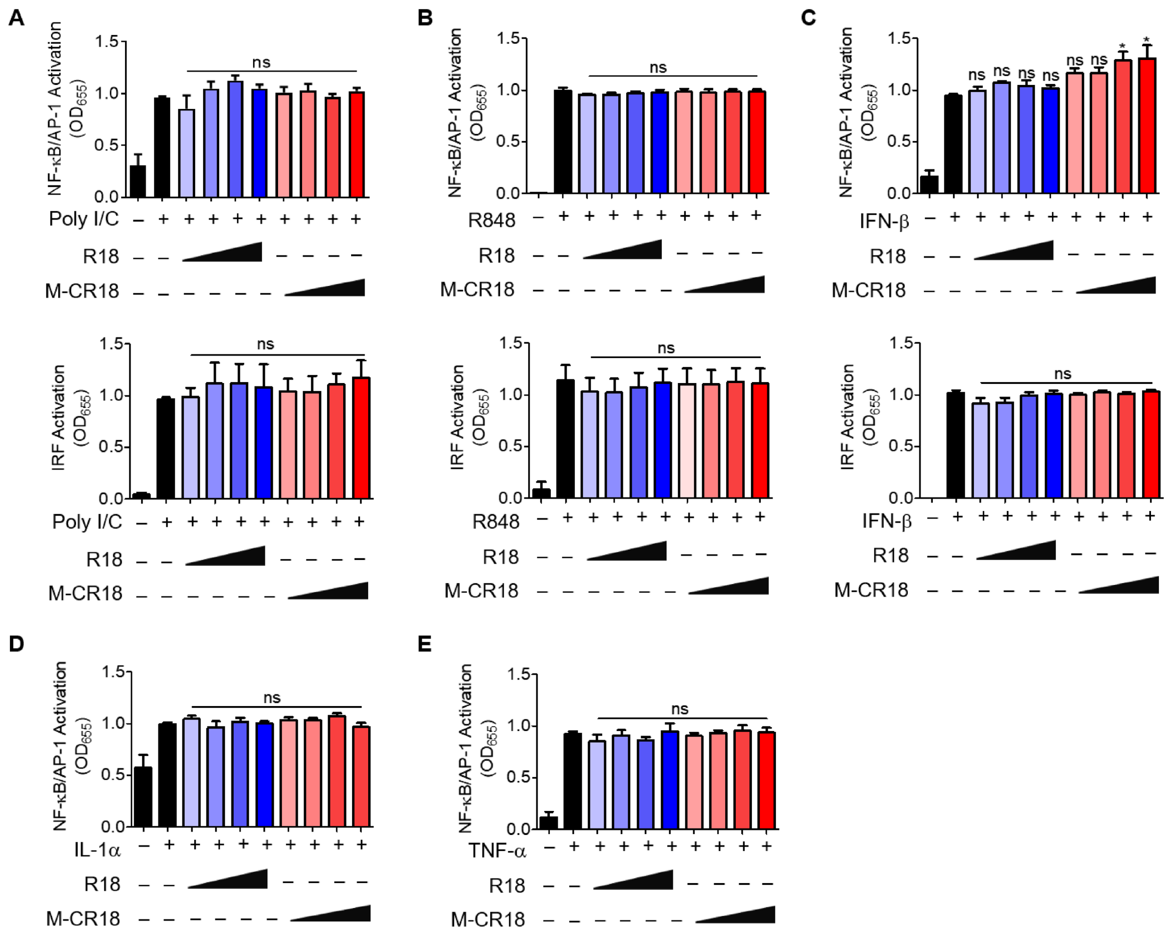

Figure S10. The effects of R18 and M-CR18 on different inflammatory signaling pathways in THP-1 reporter cell-derived macrophages. (A-C) The effects of R18 and M-CR18 on the activation of NF-κB/AP-1 (top) and IRF (bottom) upon PolyI/C (for TLR3, 50 μg/mL) (A), R848 (for TLR7/8, 10 μg/mL) (B), and IFN-β (for IFNR, 10 ng/mL) (C) stimulation for 24 h. (D, E) The effects of R18 and M-CR18 on the NF-κB/AP-1 activation under IL-1α (for IL1R, 100 ng/mL) (D) and TNF-α (for TNFR, 20 ng/mL) (E) stimulation. M-CR18 and R18 = 0.625, 1.25, 2.5, 5 μM; N = 3. The data is presented as the mean ± SEM. ns: not significant, *$p < 0.05$ vs. the stimulation group.

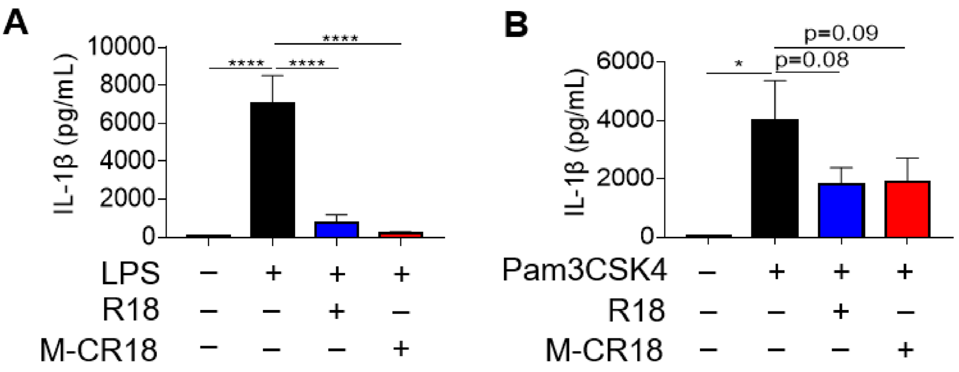

Figure S11. The effects of R18 and M-CR18 on the TLR4- and TLR2-mediated IL-1β production in THP-1 cell-derived macrophages. (A, B) The inhibition of the cytokine IL-1β production by R18 and M-CR18 under LPS (10 ng/mL) (A) and Pam3CSK4 (10 ng/mL) (B) stimulation for 24 h in THP-1 cell-derived macrophages. R18 and M-CR18 = 5 μM; N = 6. The data is presented as the mean ± SEM. *$p < 0.05$, ****$p < 0.0001$.

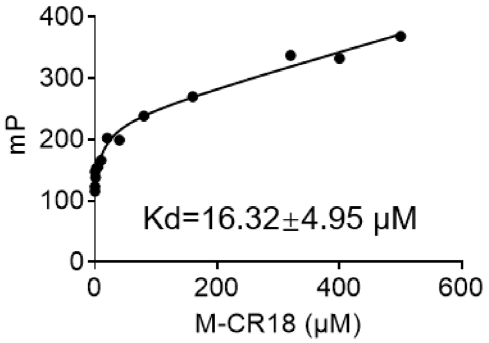

Figure S12. The binding of M-CR18 to TLR4 ligand. The fluorescence polarization profile of FITC-labeled LPS (10 ng/mL) mixed with various concentrations of M-CR18 (3.9, 7.8, 15.6, 31.25, 62.5,125, 250, 500, 1000 and 2000 μM); the dissociation constant Kd was obtained from the non-linear fitting of the curve.

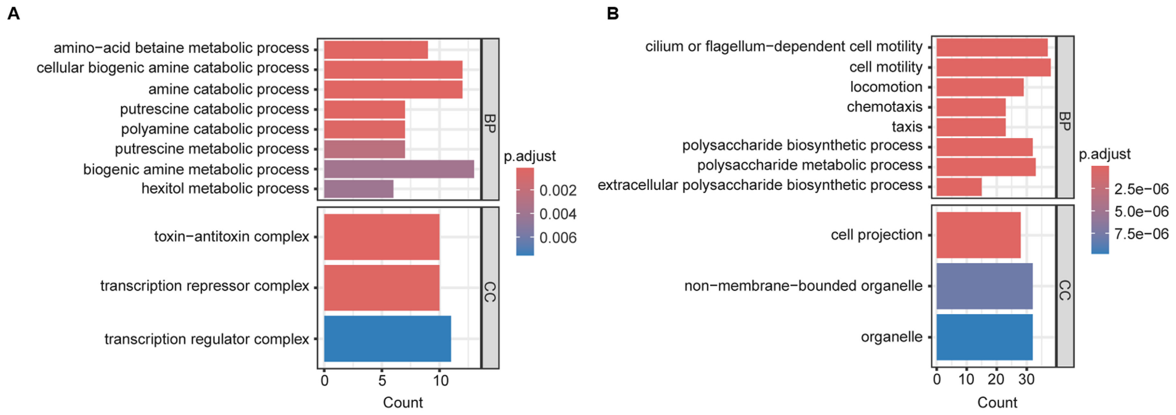

Figure S13. The enriched Gene Ontology (GO) analysis of the transcriptome of M-CR18 treated E. coli. (A) The enriched up-regulation pathways and (B) down-regulation pathways by M-CR18 compared with R18; p value < 0.05.

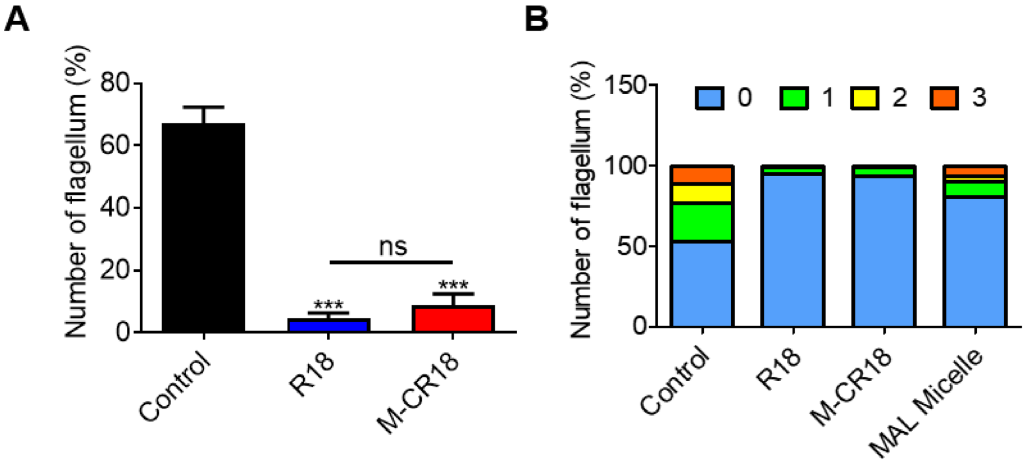

Figure S14. The effects of R18 and M-CR18 on the formation of bacterial flagella of *E. coli*. (A) The total number of bacterial flagella counted in the TEM images (Figure 6K) with or without the presence of R18 or M-CR18. (B) The counting of flagellum numbers observed in each bacterium from the TEM images (Figure 6K) in the presence of R18, M-CR18, or the MAL micelles (unmodified nanomicelles). R18 and M-CR18 = 80 μM; lipid concentration of MAL micelle = 1.25 mg/mL; N ≥ 39. The data is presented as the mean ± SEM. ns: not significant, ***p < 0.001 vs. the untreated control unless otherwise indicated.

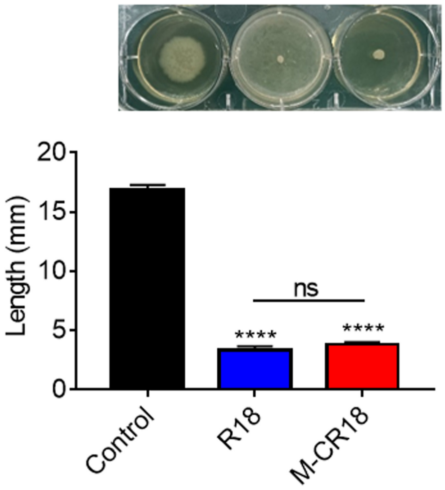

Figure S15. The effects of R18 and M-CR18 on the bacterial motility of *P. aeruginosa*. The bacterial motility of *P. aeruginosa* (PA103) was observed in the 0.5% agar plate diffusion (Top), and quantified for the colony diffusion (bottom) based on the largest distance across the colony with or without R18 and M-CR18 treatments. R18 = 200 μM, the peptide concentration for M-CR18 = 200 μM, the lipid concentration for M-CR18 = 1.25 mg/mL; N = 3. The data is presented as the mean ± SEM. ns: not significant, ****p < 0.0001 vs. the untreated control unless otherwise indicated.

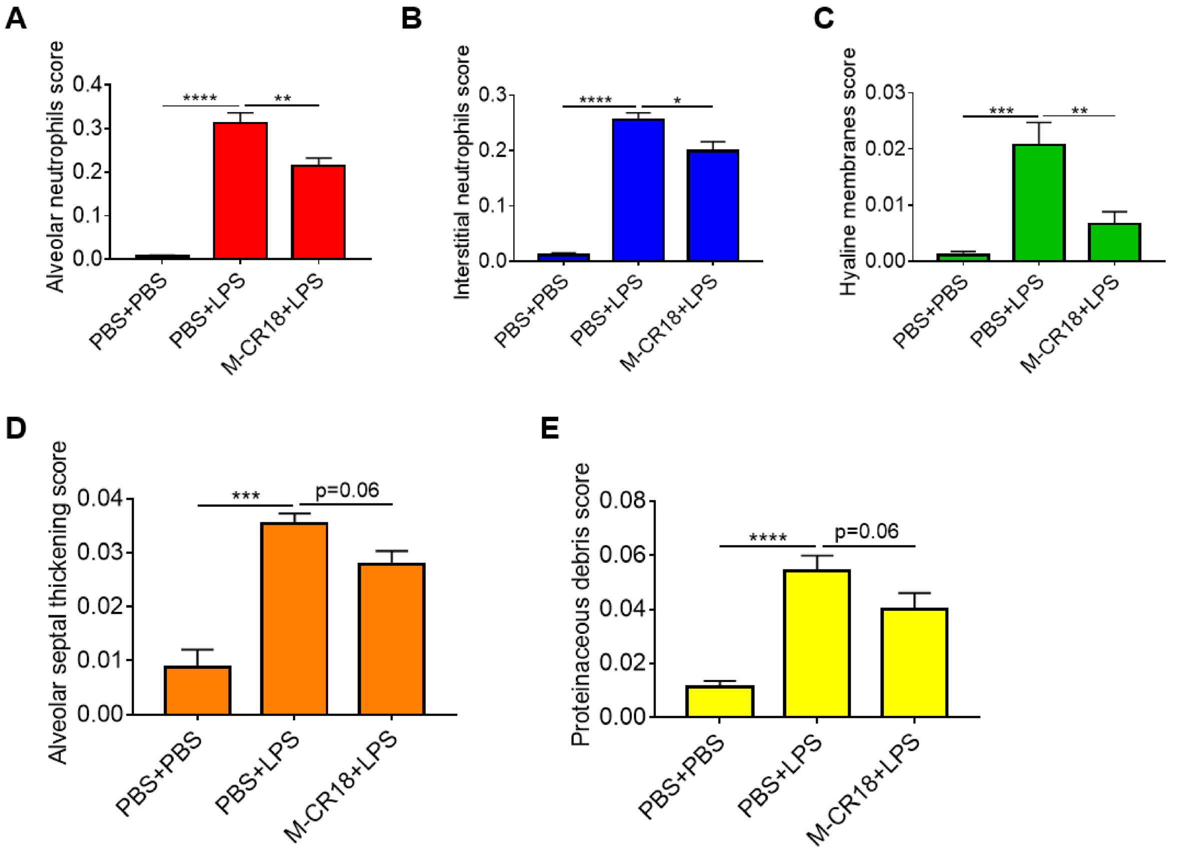

Figure S16. The scoring of five histopathological features of the injured lungs in the LPS-induced ALI mouse model. The lung injury scores of the LPS-induced ALI mice with or without the M-CR18 treatment were assessed based on the five histopathological features: alveolar neutrophil fraction (A), interstitial neutrophil fraction (B), fraction of hyaline members (C), histopathological alveolar septal thickening (D), and proteinaceous debris (E) N ≥ 5. The data are presented as the mean ± SEM. *p < 0.05, **p < 0.01, ***p < 0.001, ****p < 0.0001.

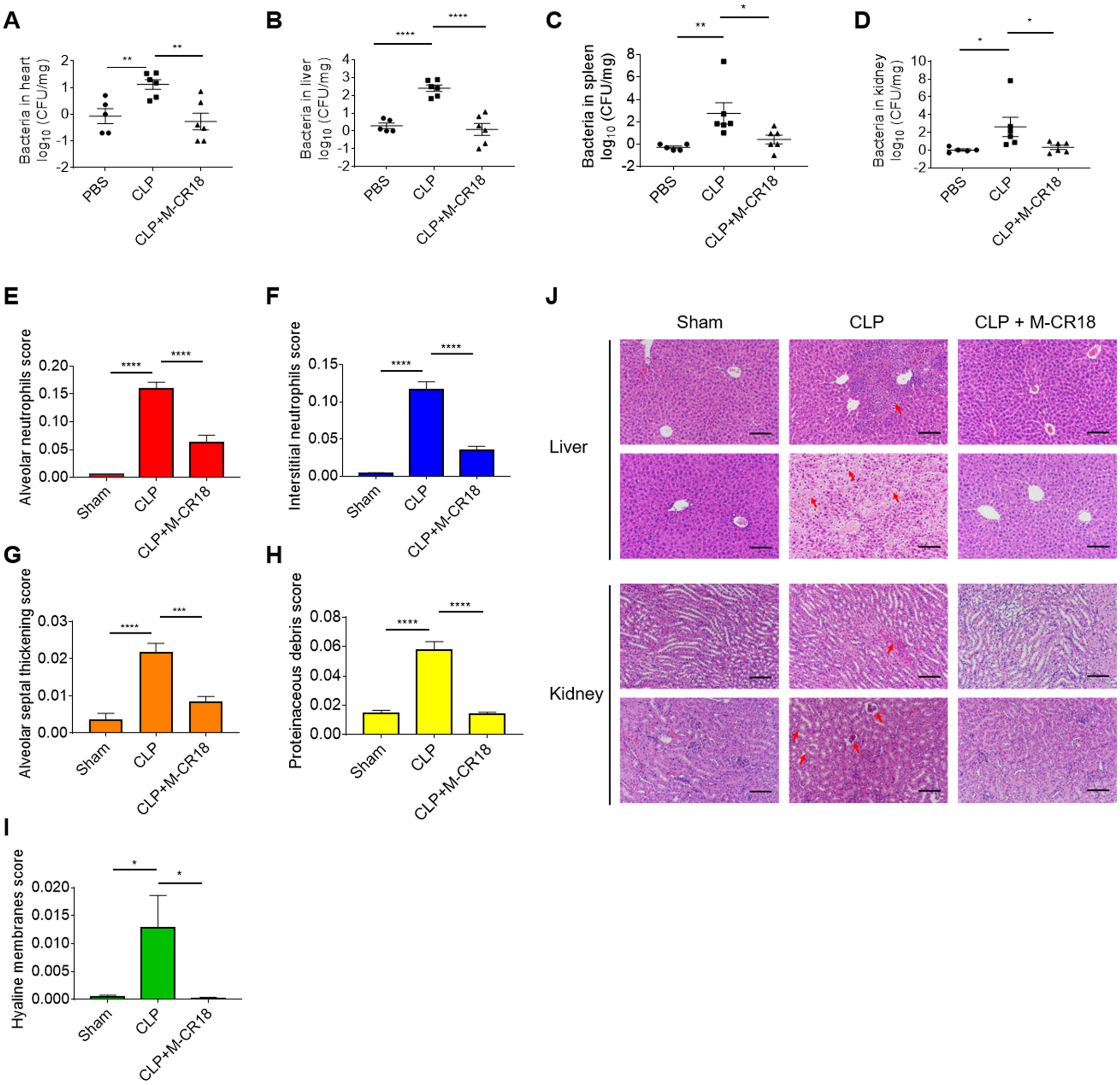

Figure S17. The effects of M-CR18 on the bacterial load in the vital organs and on the pathological scores of injured lungs in the CLP-induced mild sepsis mouse model. (A-D) The bacterial load in the heart (A), liver (B), spleen (C) and kidneys (D) of the CLP-induced sepsis mice with or without M-CR18 (120 nmol/kg) treatment; N = 6. (E-I) The histopathological scores of injured lungs based on five features: neutrophils in the alveolar space (E), neutrophils in the interstitial space (F), alveolar septal thickening (G), proteinaceous debris filling the airspaces (H), and hyaline membranes (I); $N \geq 5$. (J) The representative H&E images of the liver and kidneys of mice under CLP induction for 3 days with or without M-CR18 treatment; the red arrows indicated the site of the lesion; scale bar = 50 µm. The data is presented as the mean ± SEM. *$p < 0.05$, **$p < 0.01$, ***$p < 0.001$, ****$p < 0.0001$.

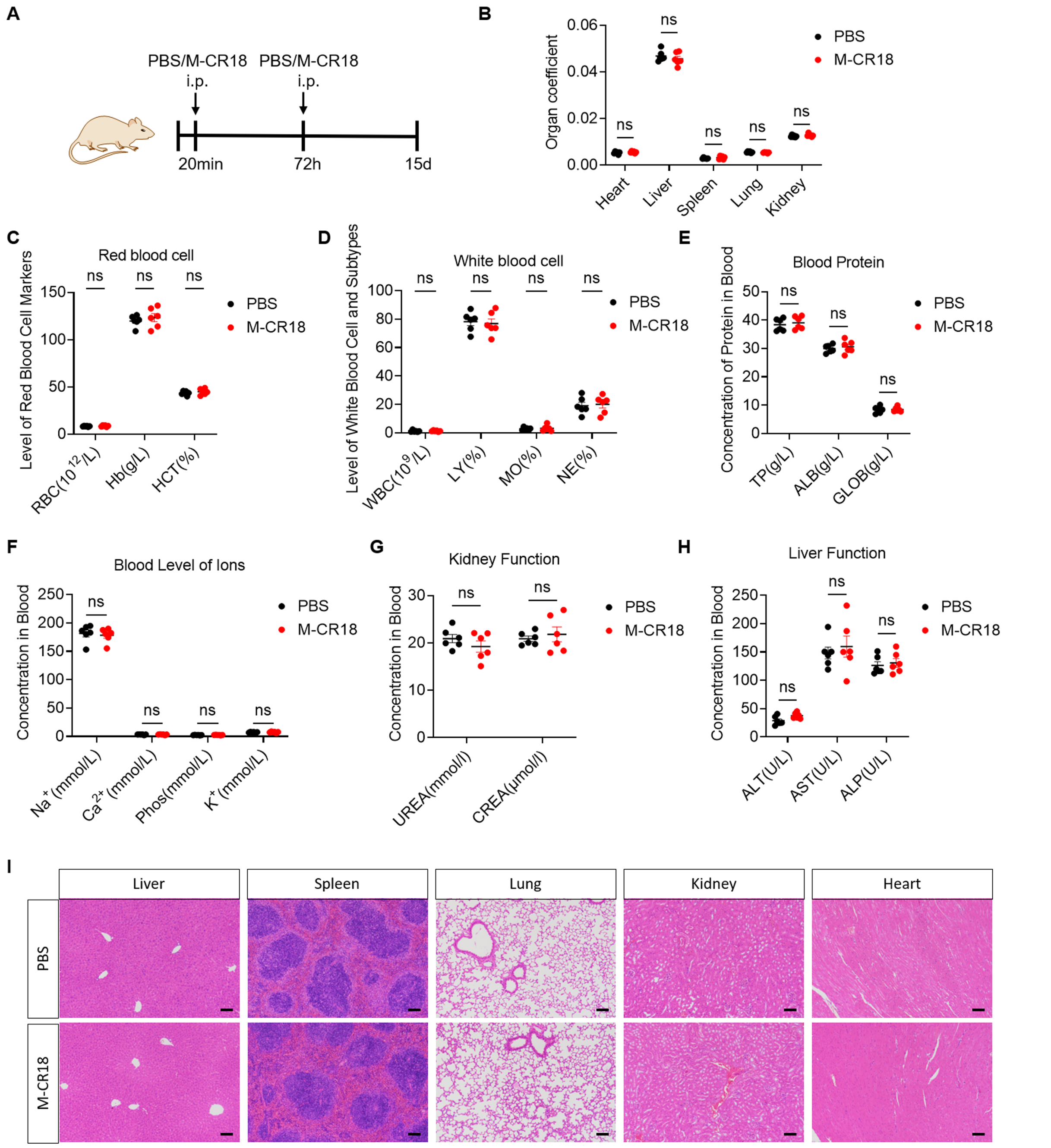

Figure S18. The biosafety profile of M-CR18 in healthy mice. (A) The schematic diagram of the biosafety test of M-CR18 treatment in healthy mice with a regime similar to the survival test of the CLP model; M-CR18 (120 nmol/kg) or PBS (same volume) was given through i.p. injection at 20 min and 72 h. (B) The weight ratio of the major organs to the whole body of the mice. (C) The levels of red blood cells (RBC), hemoglobin (Hb) and hematocrit (HCT). (D) The levels of white blood cells (WBC) and the percentage of lymphocytes (LY), monocytes (MO) and neutrophils (NE). (E) The levels of the total protein (TP), albumin (ALB) and globulin (GLOB). (F) The levels of sodium (Na^+), calcium (Ca^{2+}), phosphate (Phos) and potassium (K^+). (G) The levels of urea and creatine (CREA). (H) The levels of liver and

pancreas enzymes including alanine aminotransferase (ALT), aspartate transaminase (AST) and alkaline phosphatase (ALP). (I) Representative histological images of the H&E stained tissue sections from the liver, spleen, lung, kidneys and heart of healthy mice treated with M-CR18 or PBS; scale bar = 100 μm. The data is presented as the mean ± SEM; ns: not significant.

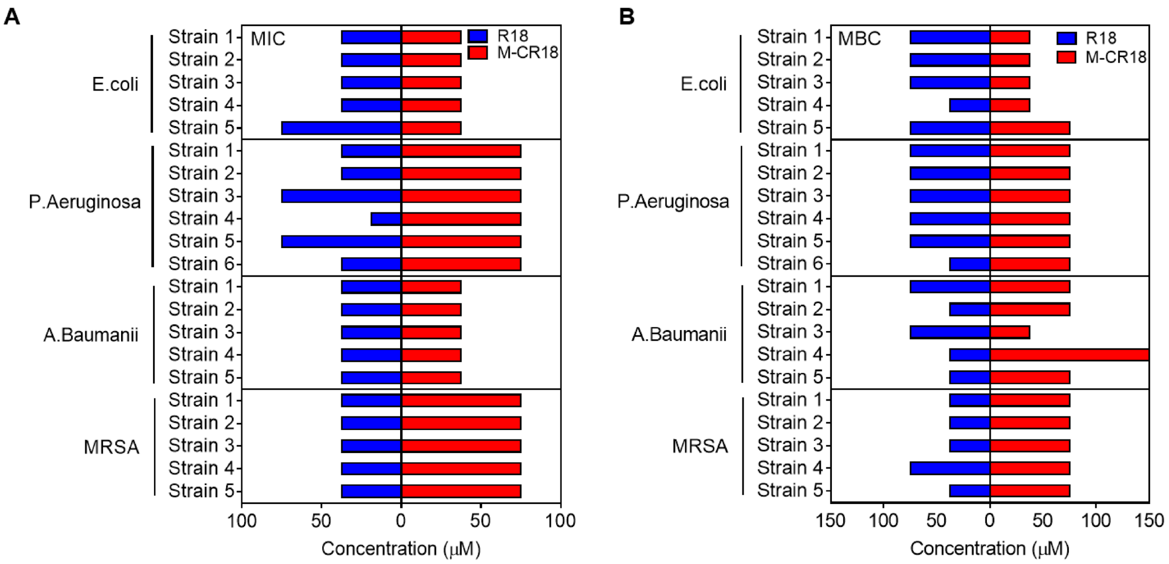

Figure S19. The effects of R18 and M-CR18 on various clinical antibiotics-resistant bacterial strains. The antibacterial ability of M-CR18 and R18 against clinically present drug-resistant bacterial strains was evaluated by the MIC (A) and MBC (B) values.

3. Supplementary Tables

Table S1. Prediction and analysis of the α-helix structure of R18.

Prediction method	α-helix (%)	Analysis methods	α-helix (%)
PredictProtein	88.9	K2D3	75.5
Chou-Fasman	83.3	BesSel	93.3
GOR	100		
Neural Network	100		

Table S2. The binding free energy ($\Delta G_{binding}$) of the specific TLR ligands or R18 to the receptors.

Receptor	Ligand	MM-GBSA $\Delta G_{binding}$ (kcal/mol)
TLR4/MD-2	Lipid A	-19.02
	R18	-36.12
TLR1/2	Pam3CSK4	-200.82
	R18	-237.76

Note: lipid A was used for the molecular docking as the LPS molecule is too large to do the calculation.

Table S3. The primer sequences used for qRT-PCR.

Primers	Sequences (5'-3')
fliA forward	TCCAGTTGCCCTATTGCCTG
fliA reverse	ATGACGCCCTACAAGGAACG
fliC forward	CAACAGCAGTCAGAGCCTCA
fliC reverse	GTCAAACTGGGCGGAGATGA
flhC forward	CAAACCGCACCAATGTCCAG
flhC reverse	CGGTGATCAAAGCCTACCGT
flhD forward	TCGTCTGGTGGCTGTCAAAA
flhD reverse	TCCGCTATGTTTCGTCTCGG
flgK forward	CCGACGAAGCGAAAATAGCG

flgK reverse	CGCCCACCGTTTTACTGTTG
flgL forward	TCGCGTTCGATGGTGATAGG
flgL reverse	CTACCGTCTGGTTCCGCTAC
rrsA forward	CGCAAGACCAAAGAGGGGTA
rrsA reverse	AGCCGTTACCCCACCTACTA
